# The genome, transcriptome, and proteome of the fish parasite *Pomphorhynchus laevis* (Acanthocephala)

**DOI:** 10.1371/journal.pone.0232973

**Published:** 2020-06-23

**Authors:** Katharina Mauer, Sören Lukas Hellmann, Marco Groth, Andreas C. Fröbius, Hans Zischler, Thomas Hankeln, Holger Herlyn

**Affiliations:** 1 Anthropology, Institute of Organismic and Molecular Evolution (iomE), Johannes Gutenberg University Mainz, Mainz, Germany; 2 Molecular Genetics and Genomic Analysis Group, Institute of Organismic and Molecular Evolution (iomE), Johannes Gutenberg University Mainz, Mainz, Germany; 3 CF DNA sequencing, Leibniz Institute on Aging–Fritz Lipmann Institute, Jena, Germany; 4 Molecular Andrology, Biomedical Research Center Seltersberg (BFS), Justus Liebig University Gießen, Gießen, Germany; University of Western Sydney, AUSTRALIA

## Abstract

Thorny-headed worms (Acanthocephala) are endoparasites exploiting Mandibulata (Arthropoda) and Gnathostomata (Vertebrata). Despite their world-wide occurrence and economic relevance as a pest, genome and transcriptome assemblies have not been published before. However, such data might hold clues for a sustainable control of acanthocephalans in animal production. For this reason, we present the first draft of an acanthocephalan nuclear genome, besides the mitochondrial one, using the fish parasite *Pomphorhynchus laevis* (Palaeacanthocephala) as a model. Additionally, we have assembled and annotated the transcriptome of this species and the proteins encoded. A hybrid assembly of long and short reads resulted in a near-complete *P*. *laevis* draft genome of ca. 260 Mb, comprising a large repetitive portion of ca. 63%. Numbers of transcripts and translated proteins (35,683) were within the range of other members of the Rotifera-Acanthocephala clade. Our data additionally demonstrate a significant reorganization of the acanthocephalan gene repertoire. Thus, more than 20% of the usually conserved metazoan genes were lacking in *P*. *laevis*. Ontology analysis of the retained genes revealed many connections to the incorporation of carotinoids. These are probably taken up via the surface together with lipids, thus accounting for the orange coloration of *P*. *laevis*. Furthermore, we found transcripts and protein sequences to be more derived in *P*. *laevis* than in rotifers from Monogononta and Bdelloidea. This was especially the case in genes involved in energy metabolism, which might reflect the acanthocephalan ability to use the scarce oxygen in the host intestine for respiration and simultaneously carry out fermentation. Increased plasticity of the gene repertoire through the integration of foreign DNA into the nuclear genome seems to be another underpinning factor of the evolutionary success of acanthocephalans. In any case, energy-related genes and their proteins may be considered as candidate targets for the acanthocephalan control.

## Introduction

Acanthocephala (thorny-headed worms) are endoparasites of jaw-bearing vertebrates (Gnathostomata) including humans [1–4]. In their digestive tract, the worms usually grow to adults of several millimeters to a few centimeters in length, followed by heterosexual reproduction. Upon insemination, the female produces large quantities of eggs containing a larval stage (acanthor). The eggs are released into the environment with the host's excrements [5] and subsequently can be orally taken up by an intermediate host from Crustacea, Hexapoda or Myriapoda [1,6]. Inside the intermediate host, the acanthor grows to a stage, which can infect the definitive host, following oral uptake of an intermediate host [7]. Additional host types may occur, but only the two-host cycle detailed above is obligatory [8].

Under unsuitable conditions acanthocephalans may penetrate the intestinal wall of their vertebrate hosts, which can elicit fatal peritonitis [9]. These migrating worms also enter mesenteries and organs such as the liver, with serious consequences for the host’s health [10]. Even if they remain in the digestive tract, thorny-head worms damage the host tissue through their usually hook-bearing attachment organ, the proboscis [11,12]. In fact, the movements of the proboscis induce bleeding, inflammatory reactions, necrosis, and lesions [13,14], which reduce the host's ability to absorb nutrients [13]. In addition, the gutless worms absorb minerals and nutrients from the blood and decaying tissue via their surface [15–18]. Considering these detrimental effects, it may surprise that high loads with acanthocephalans can be tolerated [19]. However, if further stress factors are added acanthocephalans can significantly increase host mortality [20]. Mass infections with up to ~1,500 thorny-headed worms per host can result in the death of birds, fish, etc. due to intestinal obstruction [21].

Except in the wild, acanthocephalans occur worldwide in human livestock, including domestic pigs (*Sus scrofa domestica*) and chickens (*Gallus gallus domesticus*) [16,22]. Furthermore, acanthocephalans quite regularly contribute to the parasite fauna in marine [23–25] and freshwater aquaculture [26–29], which results in growth retardation, weakening, and emaciation of fishes [30]. Thorny-headed worms are even regarded the main obstacle to successful aquaculture in some regions [14,31–36]. Despite their importance as a pest, no nuclear genome of an acanthocephalan has yet been published, nor have previous studies comprehensively captured their transcriptome and proteome, including both sexes and developmental stages. It is obvious that a better knowledge of the molecular basis of acanthocephalan pathogenicity might provide starting points for the development of new drugs to control them. In fact, due to their low specificity, the use of established anthelminthics poses risks for the environment and consumers [25,37–40], a deficit that the alternatives proposed so far do not overcome [9,30,36,41].

The study of genome and transcriptome data additionally holds the prospect of elucidating the molecular underpinnings of acanthocephalan evolution. This should be especially the case since the phylogenetic relationships of Acanthocephala are clarified in decisive points. Thus, it is generally accepted that thorny-headed worms and wheel animals or rotifers (Rotifera) are a monophyletic group referred to as either Syndermata or Rotifera, then including Acanthocephala [3,42–48]. Analyses of larger molecular datasets further revealed that rotifers in the traditional comprehension are paraphyletic. In particular, bdelloids are most probably more closely related to the acanthocephalans than to monogononts [43,49–56]. However, the last common ancestors (LCAs) of monogononts and bdelloids were probably free-living [57]. Thus, comparions with genomic and transcriptopmic data from the latter two taxa should shed light on the evolutionary changes in the acanthocephalan stem line. Along with the lifestyle, the acanthocephalan morphology underwent significant changes [2,3,45,50]. Examples for evolutionary novelties are the aforementioned proboscis and the muscular apparatus moving it [11,12]. The absent alimentary tract and the exclusive uptake of nutrients via the tegumental surface should be additional apomorphies of acanthocephalans [3,50,51,58]. In addition, the metasomal body cavity is largely committed to the production of large amounts of gametes [59]. Archiacanthocephalan females might even shed 82,000 eggs per day on average, and this for a period of 10 months [5]. Thus, a single female might produce up to 25 million eggs. Male acanthocephalans are also selected for high fertility, as illustrated by their enlarged testicles [60]. Consequently, both sexes face basically the same challenge: Ensuring high energy supply in the oxygen-depleted intestinal lumen of their vertebrate hosts [61,62]. However, the extent to which the evolutionary changes in morphology and lifestyle left signatures in the genome and transcriptome of Acanthocephala awaits clarification.

In addition, it is not clear whether the genome of acanthocephalans is more compact than that of free-living rotifers, as is known from parasitic nematodes, mites and fungi and their free-living relatives [63–66]. Another question to be clarified is if significant amounts of foreign DNA might have entered the acanthocephalan genome through horizontal gene transfer (HGT). In fact, HGT has been reported for asexual bdelloids, but not for monogononts, which sporadically reproduce sexually [67–71]. However, strict heterosexuals, such as acanthocephalans [59], have not yet been included in the comparison. If HGT occurs in acanthocephalans, it could increase their chances of adapting to the challenges of their specific lifestyle, as previously suggested for animal parasitizing nematodes [72,73], phytopathogenic nematodes [63,74,75], and parasitic plants from Orobanchaceae [76].

The above questions are addressed in this study using the first assemblies of the nuclear genome and transcriptome of an acanthocephalan. For doing so, we chose *Pomphorhynchus laevis* (Zoega in Müller, 1776) Monticelli, 1905, as a model. The species belongs to the Palaeacanthocephala, one of the acanthocephalan taxa with the traditional rank of a class [53,56,77,78]. The species measures in the range of few centimeters and parasitizes gammarids (Crustacea, Amphipoda) and ray-finned fishes [77,79,80]. Along with its hosts, *P*. *laevis* occurs in Eurasian lakes and rivers, as well as in brackish waters of estuaries and the Baltic Sea. It was also found together with anadromous and catadromous fish in the waters of the North Sea and the White Sea [77,79,81–87], thus covering the entire range of aquatic habitats known for Acanthocephala as a whole. Not least, *P*. *laevis* belongs to the best-studied acanthocephalan species in terms of morphology, ecology, life history, and pathogenicity in aquaculture [8,88–90].

## Materials and methods

### Samples

Specimens of *P*. *laevis* were excised from a common barbel (*Barbus barbus*), which was caught in June 2006 near Gimbsheim (Germany), by a fisherman under license 16692 issued by Verband Deutscher Sportfischer e. V. (VDSF). The fish was not caught for sampling but for consumption. The material used here would otherwise have been discarded. DNA and RNA samples were extracted from worms after they had freed themselves from impurities in physiological saline solution.

### DNA sequencing and preliminary assembling

Worms were decapitated and grinded, following digestion with proteinase K, DNA extraction with phenol-chloroform-isoamyl alcohol and precipitation in ethanol. Upon centrifugation, the pelleted DNA was washed in ethanol (70%) and eluted in HPLC grade H_2_O.

DNA of one *P*. *laevis* specimen was sequenced on two lanes on an Illumina HiSeq 2500 platform (100 bp, paired end, 275,633,942 reads total) by StarSEQ (https://www.starseq.com/). The quality of the raw data was checked with FastQC v.0.11.5 (http://www.bioinformatics.babraham.ac.uk/projects/fastqc/) and processed with the FastX toolkit v. 0.0.13.2 (http://hannonlab.cshl.edu/fastx_toolkit/). First, we assembled and annotated the mitochondrial *P*. *laevis* genome from these trimmed Illumina reads (see Mitochondrial genome assembly). Prior to assembling, we had removed mitochondrial genome and PhiX genomes with the kmer filtering function in BBduk (https://sourceforge.net/projects/bbmap/). Potential sequences of human (*Homo sapiens*) origin were deleted following their detection by mapping against the masked human reference nuclear genome (HG19) with BBmap (https://sourceforge.net/projects/bbmap/). Kept were 0.2% of the reads, which might have bacterial origin according to MetaCache version 0.21 [91].

The DNA of ten specimens was used for the generation of long reads using PacBio *RSII*. Two sequencing runs were carried out, with three and five SMARTcells, respectively (about 9kb insert length each, 1,091,760 reads total). We assembled the raw PacBio subreads with Canu v1.0 [92]. Using cleaned Illumina reads of one lane (L002), the raw PacBio subreads were error-corrected with Proovread version 2.14.0 [93]. The coverage of the Illumina reads used for Proovread was 40x. All other parameters were as default. For further processing, we used corrected and trimmed PacBio reads. The raw data have been deposited in the SRA database under the accession nos. SRR10569073-SRR10569081 (BioProject: PRJNA554558).

### Mitochondrial genome analysis

We built a consensus of the mitochondrial genome of *P*. *laevis* (GenBank accession no. MN5624) from parallel reconstructions based on trimmed Illumina reads. We operated MITObim [94] with a cytochrome oxidase subunit I (*cox1*) sequence of *P*. *laevis* (KF559296.1) and the mitochondrial genome of a second palaeacanthocephalan, *Leptorhynchus thecatus* (AY562383.1). The *de novo* assemblers CLC workbench 8.5.1 and Geneious R9.1 [95] were run with about 5% of the Illumina reads. The assemblies were aligned with MUSCLE [96] for manual derivation of a consensus sequence. This was first annotated with MITOS [97], followed by validation of boundaries of protein-coding genes with NCBI ORF finder (https://www.ncbi.nlm.nih.gov/orffinder/). Lastly, we employed DOGMA [98] and ARWEN [99] in addition to MITOs for tRNA gene annotation. The same annotation pipeline was applied to additional acanthocephalan mitochondrial genomes [56,78,100–104], as retrieved from GenBank: Palaeacanthocephala (*Centrorhynchus aluconis*: KT592357.1; *Leptorhynchoides thecatus*: see above; *Prosthorhynchus transversus*: KT447549.1; *Southwellina hispida*: KJ869251.1), Eoacanthocephala (*Hebesoma violentum*: KC415004.1; *Pallisentis celatus*: JQ943583.1; *Paratenuisentis ambiguus*: FR856885.2), Polyacanthocephala (*Polyacanthorhynchus caballeroi*: KT592358.1), and Archiacanthocephala (*Macracanthorhynchus hirudinaceus*: FR856886.2; *Oncicola luehei*: JN710452.1).

### *De novo* assembly of the nuclear genome

The size of the nuclear genome was estimated from k-mers (k = 21) using Jellyfish [105] and GenomeScope [106]. Following this, we employed different programs in order to create a *P*. *laevis* draft of the nuclear genome that was as coherent, complete, and of high quality as possible. This WGS project has been deposited at GenBank under accession WNNJ00000000, version WNNJ01000000.

Long reads generated with PacBio technology were assembled with Canu v1.0 [92]. For doing so, we used reads corrected with Proovread v.2.14.0 and specified 0.075 for “corrected Error Rate” and 500 for “min Overlap Length”. In a parallel approach, we assembled the filtered and trimmed reads from Illumina sequencing with Platanus v.1.2.4 [107]. The best Platanus assembly, selected according to the parameter N50 (k = 21; s = 2), and Proovread-corrected PacBio reads were hybrid-assembled with DBG2OLC v.1.0 [108]. Subsequently, we combined the Canu assembly (corrected PacBio) and DBG2OLC assembly (corrected PacBio+Illumina) with Quickmerge [109]. We ran Quickmerge with stringent parameters for contig merging (HCO 6, -C 2), with the Canu assembly as a reference or donor assembly. We then employed the scaffolding function in PBJelly v.15.8.24 [110] to fill as many of the remaining gaps between contigs with the long Proovread-corrected PacBio reads (nCandidates = 20). Contigs of < 1,000 bp after PBJelly scaffolding were excluded from further processing. Finally, the contigs were corrected with Proovread. For this correction step, the trimmed and filtered Illumina reads of one lane (L002) were used again. The coverage of the Illumina reads was given as 40x; all other parameters were as default. Illumina and PacBio reads were mapped to the final draft genome using the mem algorithm of BWA v.0.7.15 [111].

We used BUSCO v.3.0.1 [112] to assess the completeness of the *P*. *laevis* nuclear genome draft. From the alternatives tested, including gene models of the hexapod *Drosophila melanogaster* and the nematode *Brugia malayi*, *Schistosoma mansoni* (Platyhelminthes, Trematoda) gene models allowed the detection of the highest number of genes by the prediction program AUGUSTUS [113] implemented in BUSCO. Since the relaxation of the TBLASTN E-value or the training of AUGUSTUS with *P*. *laevis* ESTs [50] did not lead to a significant improvement, we finally applied *S*. *mansoni* gene models. For doing so, we operated BUSCO with standard settings for E-value and activated the AUGUSTUS optimization mode for self-training. For comparison, we used the same settings for re-analysis of the nuclear genome of the bdelloid *A*. *vaga* [67].

To the best of our knowledge, no barbel genome or transcriptome was published at the time of the study. Thus, to detect potential fish contamination, we compared all reconstructed contigs to the closest phylogenetic relative of barbel, for which corresponding data was available, i.e. the common carp (*Cyprinus carpio*) (see [114] for a cyprinid phylogeny). For comparison, we analyzed a custom database with genomic DNA and transcripts from BioProject PRJNA352247, mitochondrial DNA (AP009047.1; [115]), and TSA data (GFWU01000001-GFWU01049434) with NCBI BLAST+ [116] (MEGABLAST, E-value: 1e-05; Ident: 85%). Given an average sequence divergence of approximately 15% between carp and barbel (see Fig 1 in [114]), we considered BLAST hits of ≥ 85% identity as indicative of potential contamination. However, such hits in the reconstructed contigs could alternatively represent highly conserved homologues or HGT candidates in the *P*. *laevis* nuclear genome. We therefore kept such sections in the genome assembly, summarized the MEGABLAST results in the supplements (S1 Table), and quantified their share to the nuclear genome assembly.

### Annotation of repetitive DNA and tRNA genes

We searched the *P*. *laevis* nuclear genome draft for tRNA genes with the program tRNAscan-SE 2.0 [117] and annotated repeats with RepeatMasker version open-4.0.7 [118], specifying the slow search option. For *de novo* generation of a custom database of *P*. *laevis* repeats, we ran RepeatModeler [119] on the draft genome and dnaPipeTE [120] on all trimmed and filtered Illumina Reads. As recommended [121], the best-fitting coverage depth (0.01) was initially determined by parallel runs of dnaPipeTE (N = 50) with alternative settings (for details, see S.3). Contigs with at least 90% identity were clustered with CD-HIT-EST [122]. Subsequently, processed Illumina reads of one lane (L002) were mapped with the mem algorithm of BWA v.0.7.15 to cluster-specific main sequences. We annotated clustered contigs of at least 100 bp with TEclass [123], provided their coverage was at least three times as high as the minimum average genome coverage. To avoid the masking of duplicated protein-coding genes, which were not derived from transposable elements, corresponding candidates were removed from the repeat database. For this purpose, we excluded contigs showing BLASTX hits (E-value: 1e-05) to the Swiss-Prot database (release 2018_10) [124], if the Swiss-Prot sequences lacked significant matches in RepBaseRepeatMaskerEdition-20181026 (TBLASTN; E-value: 1e-05). Finally, we added repeats from the RepBaseRepeatMaskerEdition-20181026 classified as root, Metazoa, Protostomia, or Rotifera to the custom repeat database. Despite the measures taken for avoidance, highly derived protein-coding sequences of *P*. *laevis* could have remained erroneously in the repeat database, thus overestimating the repetitive portion of the nuclear genome. For assessing the extent to which this might have occurred, we analyzed how many transcript reads mapped to the unmasked genome draft but not to the masked one (see RNA-seq *de novo* assembly). This was done with GMAP version 2018-07-04 [125] using default settings.

### RNA sequencing (RNA-seq)

To cover as many transcripts as possible, we pooled RNAs of two females, two males, and two juveniles. The respective *P*. *laevis* specimens were all collected from the same *B*. *barbus* individual, from which the worms for DNA analysis were excised. For RNA extraction, we used the TRI Reagent™ protocol (Invitrogen™). Pelleted RNA was resolved in HPLC grade H_2_O. Following poly-A capture and library construction, RNA-seq was carried out on an Illumina NextSeq platform (150 bp, paired end, 37,016,182 reads total) by StarSEQ. The raw data have been deposited in the SRA database under accession no. SRR10344638 (BioProject: PRJNA554558). Following examination with FastQC v0.11.5, we trimmed the raw reads with Trimmomatic v.0.36 [126]. Screening of the reads with MetaCache version 0.21 confirmed high purity of the samples: only 0.4% of the reads were of potential bacterial origin.

### *De novo* assembly of a reference transcriptome

This TSA project has been deposited at GenBank under accession GIBA00000000, version GIBA01000000, following routine check by NCBI. We assembled the *P*. *laevis* transcriptome from trimmed RNA-seq reads with the aid of Trinity v2.4.0 [127], using standard parameters, except for omitting read normalization. BLAST+ hits (MEGABLAST, E-value: 1e-05) of Trinity transcripts to NCBI’s Human genomic + transcript database (update date: 28.03.2018) led to removal, when the identity to the matched human sequence was ≥90% and query coverage exceeded 50%. Furthermore, we compared the transcripts to the custom *C*. *carpio* database (see *De novo* genome assembly; S2 Table). Transcripts were mapped to the nuclear genome draft of *P*. *laevis* to achieve an approximate map of the coding sequences. For this purpose, the assembled mitochondrial *P*. *laevis* genome was added to the dataset. Transcript mapping was done with GMAP version 2018-07-04. We translated the transcripts into proteins with TransDecoder v5.2.0 [128]. Only single best proteins (per transcript) of at least 30 amino acids (aa) were issued. The transcriptome-derived *P*. *laevis* proteome was checked with BUSCO v.3.0.1 for completeness.

### Transcript annotation and analysis

We annotated the transcripts with the aid of the Trinotate v3.1.1 pipeline (https://github.com/Trinotate/Trinotate.github.io/wiki), using various methods and databases. Thus, transcripts and TransDecoder open reading frames (ORFs) of 30 codons or more were blasted against the Swiss-Prot database (release 2018_10) with NCBI BLAST+. In the case of hits (E-value < 1e-03), Trinotate collected the corresponding gene ontologies (GOs). The distribution of second level GO terms covering at least 1% of the transcripts was visualized with WEGO [129]. We also compiled a custom database composed of all NCBI proteins from Ancylostomatidae, Ascarididae, *Dracunculus*, Filariidae, Oxyuridae, and Strongylidae (all Nematoda), as well as from Cestoda, Digenea, Monopisthocotylea, Polyopisthocotylea, and *Schistosoma* (all Platyhelminthes), besides Acanthocephala and (other) Rotifera. After removing identical sequences, the database contained 317,929 proteins. Following the annotation, we checked the transcripts for mitochondrial content and confirmed *P*. *laevis* as the sequenced species by BLASTN of the *cox1* sequences.

For the detection of orthologous proteins in *P*. *laevis* and rotifers, we translated the previously published transcriptomes of the monogonont *Brachionus manjavacas* (GFGK01000001-GFGK01065541; [130]) and the bdelloid *Rotaria magnacalcarata* (GDRE01000001-GDRE01037876; [131]) with TransDecoder, as done before for *P*. *laevis*. Orthologous clusters were then identified with OrthoVenn1 [132]. The according server uses a modified version of OrthoMCL [133] for the clustering of orthologous proteins and then annotates the clusters searching the non-redundant UniProt database with BLASTP [116]. OrthoVenn also conducts GO term enrichment analyses for species-specific and shared groups of orthologous proteins. In detail, we specified the metazoan database, uploaded the protein sets of *P*. *laevis*, *B*. *manjavacas*, and *R*. *magnacalcarata* and ran the program with default settings.

In addition, we calculated p-distances from single-copy orthologues shared across species, which had been aligned with mafft version 7.427 and pruned from uncertain alignment sections with Gblocks version 0.91b [134]. The latter sotware was run with default settings, except for a lowered minimum block length (5 aa). We then used MEGA X [135] to infer p-distances from pairwise comparisons of orthologues of *B*. *manjavacas*, *R*. *magnacalcarata*, and *P*. *laevis*, assuming a uniform substitution rate. Subsequently, we tested for equality of the p-distance levels with the Kruskal-Wallis test in SPSS version 23.0 (IBM). Median p-distances were used for depiction of phylogenetic trees with MEGA X (neighbor-joining).

The same single-copy clusters were independently divided by two persons into alternative functional classes according to their OrthoVenn annotations. Transcripts with direct involvement in energy metabolism (e.g. ATP synthase subunit alpha, mitochondrial, Glycogen phosphorylase 2, and NADH dehydrogenase [ubiquinone] flavoprotein 2, mitochondrial) were grouped in one class. In the other class, we combined transcripts, for which an involvement in energy metabolism was not evident. Transcripts with uncertain classification or lacking an annotation were not further considered (S3 Table). The remaining coding sequences (CDSs) were aligned and curated with pal2nal version 14.0 [136], for maintenance of codons. The alignments generated of each class were subsequently concatenated, followed by inference of values for p-distance and the rate ratio of non-synonymous to synonymous substitution rates (dN/dS) with codeml in PAML v.4.9j [137] from pairwise comparisons of the concatenated sequences.

In order to determine potential HGT from non-metazoans, translated proteins of *P*. *laevis*, *B*. *manjavacas*, and *R*. *magnacalcarata* proteins were blasted against the protein database Uniref90 with DIAMOND [138] (500 hits, E-value: 1e-05). Applying the script of Nowell et al. [68], we defined proteins with HGT index > 30 and a consensus hit support > 90% as HGT candidates. We excluded hits referring to acanthocephalans and (other) rotifers. Transcripts, from which HGT candidates were derived, were finally tested for possible origin from contamination with foreign tissue. For this purpose, we compared corresponding candidates with the NCBI non-redundant database (download at 03-07-2019) with NCBI BLAST+ MEGABLAST. We then deleted each transcript showing a hit to a non-rotifer/non-acanthocephalan sequence with an E-value ≤ 1e-05 and an identity ≥ 85%. In addition, we built a BLAST database of *P*. *laevis* HGT candidates, in order to compare them to candidates from *B*. *manjavacas* and *R*. *magnacalcarata* via BLASTP.

## Results and discussion

### Mitochondrial genomes of *Pomphorhynchus laevis* and other acanthocephalans

**Fig 1:**
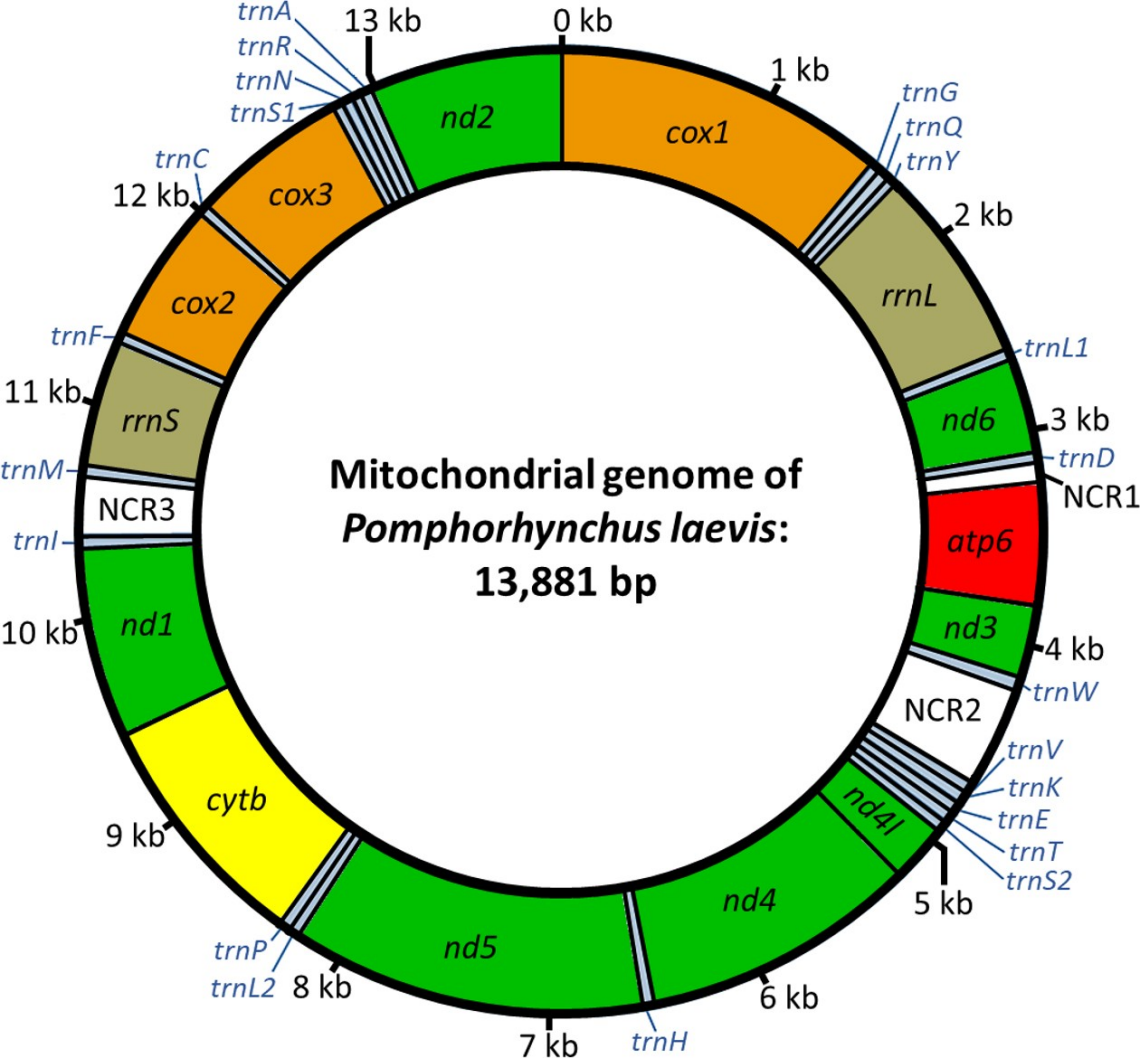
Schematic depiction of the annotated mitochondrial genome of *P*. *laevis*. The mt genome has a total length of 13,881 bp and contains 12 protein-coding genes (in alphabetical order): *atp6*: ATP synthase subunit 6; *cox1-3*: cytochrome c oxidase subunits 1–3; *cytb*: cytochrome b; *nd1-6*: NADH dehydrogenase subunits 1–6. The genes *rrnS* and *rrnL* code for 12S and 16S rRNA, respectively. Blue highlights tRNA genes (*trn*) for the twenty canonical amino acids, which are given in one-letter code (e.g. *trnA*). tRNA genes for serine (*trnS*) and lysine (*trnS*) have two copies, each. Two non-coding regions (NCR) are shown in white. The sequence is available at GenBank under accession no. MN562482. *kb*, kilobase.

The mitochondrial genome of *P*. *laevis* extends over 13,881 bp and contains the typical metazoan set of 36 genes on the heavy strand (Fig 1). Comparison of the *cox1* sequence with GenBank entries confirmed *P*. *laevis* as the sequenced species [139]. As in other Gnathifera [140,141], thereunder acanthocephalans [56,101,102], *atp8* was not annotated in the *P*. *laevis* mitochondrial sequence. Also, gene order was very similar in *P*. *laevis* and other acanthocephalan species. In particular, protein-coding genes and ribosomal rDNAs were identically arranged in *P*. *laevis* and ten re-analyzed mitochondrial genomes from Archiacanthocephala, Eoacanthocephala, Palaeacanthocephala, and Polyacanthocephala (see Materials and methods for details). Differences in mitochondrial gene order between *P*. *laevis* and the other species were confined to single tRNA genes. However, the annotation of tRNA genes is known to be challenging, due to their higher substitution rates and sporadic degeneration of secondary structure [51,98,142]. Nevertheless, we observed identical synteny in *P*. *laevis* and another palaeacanthocephalan, *L*. *thecatus* [103], and in the archiacanthocephalans *M*. *hirudinaceus* [78] and *O*. *luehei* [100]. We additionally detected alternative (GTG, TTG, ATT, ATA) and incomplete stop codons (T) in protein-coding genes as previously reported for the mitochondrial genomes of other acanthocephalan species [56,101–103]. Moreover, with 42.5%, the GC content of the *P*. *laevis* mitogenome was in the range of other acanthocephalan and rotifer mitogenomes [56,103,104,141,143].

### Comparative analysis of the nuclear genome

Contamination of the sequenced acanthocephalan sample with host DNA should not have played a major role in the present assembly, if it occurred at all. In fact, only 4% of the contigs matched with sequences in the combined genome and transcriptome database of carp as the closest phylogenetic relative of barbel [114], from which the analyzed *P*. *laevis* specimens originated (for details, see Materials and methods). However, the average alignment length of these BLAST hits was only 75 bp (maximum 345 bp) and all hits together amounted to only 0.01% of the total assembly size. As these hits could represent conserved sequence motives, we kept them in the assembly (for BLAST results, see S1 Table).

With ca. 260 Mb (Table 1), the total span of the haploid genome draft of *P*. *laevis* was very close to the GenomeScope prediction of 265–281 Mb. In further support of its near completeness, more than 98% of the genomic Illumina reads and 99.4% of the Proovread-corrected PacBio reads mapped back to the genome draft. This corresponds to an average coverage of 81x and 17x by the Illumina and PacBio reads, respectively, so that a total coverage of 100x was approximately reached. Thereby, the current draft genome of *P*. *laevis* consists of 4,021 contigs of 65 kb on average (contig N50 = 126,104 bp), making it one of the most coherent within the Rotifera-Acanthocephala clade (Table 1). Only the draft genomes in *Brachionus* calyciflorus, *Brachionus koreanus* and the *Brachionus plicatilis* species complex are less fragmented, but this coincides with either a much smaller size of the assembly (51 Mb [144], 85 Mb [145]), or the introduction of 6.41% [146] and 5.26% [147] of ambiguous bases (Ns) in the process of scaffolding. In contrast, we decided against such procedure and present a draft genome of *P*. *laevis* without any N.

**Table 1:** Metrics of the *P*. *laevis* draft genome in comparison to monogonont and bdelloid rotifers.

Key parameters of assemblies	Monogononta *B*. calyciflorus*	Bdelloidea *A*. vaga*	Acanthocephala *P*. laevis**
Size [bp]	129,636,934	217,933,776	260,316,196
No. contigs	9,394	41,968	4,021
Contig N50 [bp]	26,524	94,665	126,104
No. scaffolds	1,041	36,167	-
Scaffold N50 [bp]	786,674	260,259	-
GC content [%]	24.2	31.2	32.9
N content [%]	6.4	1.9	0

* according to [67,146]

**, newly generated data.

The total size of the *P*. *laevis* draft genome ranges within according estimates for other parasitic taxa such as Platyhelminthes (104–1,259 Mb) and Nematoda (42–700 Mb) [148]. The nuclear genome size additionally meets the expectations obtained from closer phylogenetic relatives of acanthocephalans, i.e., bdelloids and monogononts [67,68,146,149]. According to flow cytometry measurements, for example, haploid genomes should have about 117–225 Mb in the monogonont genus *Brachionus* (1 pg = 978 Mb; [150,151]). Compared to monogononts, bdelloids seem to have larger nuclear genomes, which probably reflects their tetraploid nature [67,152,153]. In fact, cytofluorometric measurements suggest that *Adineta vaga* and *Philodina roseola* have haploid genomes of about 245 Mb and 1,193 Mb, respectively (1 pg = 978 Mb; [151,154]). However, more revealing should be the comparison with size estimates of haploid genomes according to *de novo* assemblies, which span 129.6 Mb in *B*. *calyciflorus* and 51–115 Mb in the *B*. *plicatilis* species complex [144,146,149]. The corresponding estimates for bdelloids are in a higher range once more, with up to 217.9 Mb (*A*. *vaga*), 201.3 Mb (*Adineta ricciae*), 295.4 Mb (*Rotaria macrura*), and 337.6 Mb (*R*. *magnacalcarata*) [67,68]. Thus, the size of the *P*. *laevis* genome is within the limits of bdelloid genomes, while monogononts have smaller genomes. Likewise, the GC content of the *P*. *laevis* draft genome (32.9%) is very similar to corresponding values in the bdelloids *A*. *vaga* (31.2%), *A*. *ricciae* (35.6%), *R*. *magnacalcarata* (31.9%), and *R*. *macrura* (32.6%), while GC contents are clearly lower in the monogononts *B*. *calyciflorus* (24.2%) and *B*. *plicatilis* (26.4%) [67,68,146,149]. As long as corresponding findings for other gnathiferan taxa like Gnathostomulida and Micrognathozoa [54,155,156] are not available, ancestral conditions remain uncertain. However, current evidence does not contradict a closer phylogenetic relationship of bdelloids with acanthocephalans than with monogononts [49–52].

### Repeats, tRNA genes, and the non-repetitive portion in nuclear genomes

With 63%, repetitive elements recognized by RepeatMasker make up a larger part of the draft genome in *P*. *laevis* than published for traditional rotifer taxa. Thus, RepeatMasker estimates for the repetitive portion in *de novo* assemblies of monogonont and bdelloid genomes do not exceed 28% [68,146]. This discrepancy could reflect an overestimation of the repetitive portion in the *P*. *laevis* genome, and indeed GenomeScope gives a lower range between 45% and 51%. However, there may also be an underestimation in respect to the nuclear genomes of traditional rotifer taxa (see also [157]). In support of such a possibility, dnaPipeTE detected a repeat portion of up to 44% in *Brachionus asplanchnoidis* [144]. In any way, the non-repetitive portion of the *P*. *laevis* draft genome spans ca. 35%. This corresponds to 96.3 Mb, which is close to the size of the non-repetitive part in nuclear genomes of traditional rotifers as exemplified by about 102.3 Mb in *B*. *calyciflorus* [146]. Thus, variation of nuclear genome size in the Rotifera-Acanthocephala clade appears to be mainly due to the plasticity of the repetitive fraction.

A greater repetitive fraction in the nuclear genome of *P*. *laevis* is also evident in respect to protein-coding genes. In detail, 27% fewer Trinity transcripts mapped to the masked genome than to the unmasked one (for GMAP results, see S4 Table). This corresponds to 11,787 transcripts, of which the Trinotate v3.1.1.1 pipeline (via BLASTX or BLASTP) annotated 1,207. The majority of the annotated transcripts, about 57%, referred to transposon activity, for which repetition is to be expected, such as RNA-directed DNA polymerase from mobile element jockey and Retrovirus-related polyprotein from transposon Gypsy (S5 Table). According to RepeatMasker, Long Interspersed Nuclear Elements (LINEs) occupied the largest fraction (36.01%) amongst the repetitive elements in the *P*. *laevis* genome (S.3). This was followed by unclassified repeats (16.20%), Long Terminal Repeats (LTRs: 5.54%), DNA transposons (2.94%), and Short Interspersed Nuclear Elements (SINEs: 0.38%). The low proportion of SINEs in the *P*. *laevis* genome is especially surprising considering the proven expression of RNA-directed DNA polymerases (for Trinotate annotation, see S7 Table). On the other hand, low representation of SINEs seems to be common in the Rotifera-Acanthocephala clade. In *B*. *calyciflorus* and *A*. *vaga*, for example, SINEs make up only 0.00–0.08% of the repeats [144,146].

Using tRNAscan-SE, we additionally detected 551 tRNA-coding sequences in the *P*. *laevis* draft genome, 29 of which were classified as pseudogenes (S6 Table). Most tRNA genes in the *P*. *laevis* genome transferred cysteine, followed by tRNAs for glutamine and glycine. Frequently, several copies of the same tRNA gene occurred in tandem, with only small distances between them. For instance, one contig (Contig3391) contained 57 cysteine tRNA genes separated by 76 to 1,253 base pairs (S6 Table). In any case, the number of tRNA genes in *P*. *laevis* was within the range of 543–984 tRNA genes reported for bdelloids [68]. In contrast, the corresponding count (1,063 tRNA genes) in the monogonont *B*. *calyciflorus* was clearly higher [146]. Thus, bdelloids are more similar to acanthocephalans in terms of the number of tRNA genes than to monogononts, just as mentioned above in respect to genome size and mitochondrial GC content.

### Transcriptome and proteome analyses

The Trinity assembly contained 43,075 transcript contigs, 64 of which had MEGABLAST hits (E-value ≤ 1e-05, ≥ 85%) to the common carp database. However, the alignments had an average length of only 69 bp (maximum 611 bp), and at least some of them could be related to HGT events. Once more, we did not delete sequences with matches to carp but reported them (S2 Table). But we have again filtered against potential origin from other organisms, including humans (for details, see Materials and methods). This reduced the number of remaining contigs to 42,888, totaling 33,776,651 bp (Table 2). Trinity grouped these into 28,798 gene clusters based on common sequence contents. The lower number of Trinity clusters probably reflects the occurrence of alternative splicing and paralogues in *P*. *laevis*. In any case, the combination of RNAs from two adult males, two adult females, and two juveniles obviously led to a near-to complete transcriptome assembly. In addition, 91% of the reads could be mapped to the draft genome, when applying a quality cutoff of 90% identity and 50% query coverage. The percentage was even 96%, when GMAP was run with default settings (S4 Table).

**Table 2:** Transcriptome and proteome metrics of *P*. *laevis* and monogonont and bdelloid rotifers.

Key parameters of assemblies	*Monogononta B*. manjavacas*	Bdelloidea *R*. magnacalcarata*	Acanthocephala *P*. laevis**
Transcriptome length [bp]	40,097,144	30,999,243	33,776,651
No. of contigs	65,541	37,876	42,888
Contig N50 [bp]	786	1,081	1,374
Mean contig length [bp]	612	818	788
Longest contig [bp]	12,116	6,238	15,909
Shortest contig [bp]	200	200	200
No. of proteins	35,747	35,161	35,622
Proteome lengths [aa]	5,789,655	7,539,034	7,059,720
Mean protein length [aa]	162	214	198
No. of OrthoVenn1 clusters (included proteins %)	6,068 (28.7%)	8,768 (58.8%)	6,546 (58.0%)

* re-analyzed data [130,131]

** newly generated data.

In support of the occurrence of alternative splicing, the *P*. *laevis* transcript contigs, which had passed the filtering steps, coded for 35,622 proteins, according to TransDecoder (minimal size: 30 aa). This roughly corresponds to the 35,161 and 35,747 proteins, which the same pipeline detected in the transcriptome drafts of the monogonont *B*. *manjavacas* [130] and the bdelloid *R*. *magnacalcarata* [131], respectively. Nevertheless, the difference was smaller between the bdelloid and *P*. *laevis* than between the acanthocephalan and the monogonont, which agrees with the phylogeny [43,50–52]. We additionally found the total extension of the proteome to be approximated between the bdelloid and *P*. *laevis*, while it was clearly lower in the monogonont. Also, the average protein length in the bdelloid *R*. *magnacalcarata* and the acanthocephalan *P*. *laevis* was similar (Table 2).

### Metazoan genes in acanthocephalan evolution

About 30% of the *P*. *laevis* transcripts were found to have counterparts in a custom database, in which mainly other parasitic taxa and rotifers were represented (see Materials and methods). In addition, 29% of our transcriptome data could be matched to the Swiss-Prot database by Trinotate, whereby 28% could be functionally annotated (S7 Table). Thus, about one third of the *P*. *laevis* proteins should be of higher phyletic age at minimum. Compared to this, the proportion of particularly important metazoan genes conserved in *P*. *laevis* is higher. In detail, 61% of the genes (including fragmented ones: 6.9%) contained in the BUSCO gene set for Metazoa were represented in the *P*. *laevis* draft genome. But this percentage was probably an underestimation (compare [112]), due to the non-recognition of highly derived genes. In line with this, BUSCO recognized a much higher number of 739 metazoan genes (including fragmented ones: 3.1%), corresponding to 75.5%, when operated with translated transcripts, which display the non-synonymous nucleotide substitutions only. Still, 15 genes were exclusively determined in the draft genome. These genes might have been expressed at a very low level in the analyzed *P*. *laevis* specimens. In any case, a total of 754 (739+15) and thus 77% of the functionally important metazoan genes were found conserved in *P*. *laevis*.

The reduced number of usually conserved metazoan genes presumably reflects gene loss on the lineage to *P*. *laevis*, as it is common in parasite evolution [63,158–160]. This is indeed very likely considering that almost identical percentages of metazoan genes (74% to 77%) were detected by BUSCO in the genome assemblies of four parasitic trematodes [161]. Although these counts refer to the subset of usually conserved metazoan genes, the relation could apply to the entire gene repertoire, as illustrated again by other taxa. Thus, the parasitic nematode *Trichinella spiralis* has about 22% less genes than the free-living model *Caenorhabditis elegans* [162]. In acanthocephalans, a reduced gene repertoire might reflect their simplified anatomy, as exemplified by the lacking digestive tract [3,50,163]. Genes controlling the differentiation of the digestive tract in other taxa should thus be candidates for gene loss in acanthocephalans. In line with the postulate, anterior class *hox* genes (paralogues of *hox1* and *hox2*), which control the development of the stomatogastric nervous system in other species of Gnathifera and Lophotrochozoa [155,164], could not be detected, neither in the transcriptome nor in the draft genome of *P*. *laevis*. Still, some of the genes might not have been detected in *P*. *laevis* as well as other parasites.

BUSCO classified 5.3% of the metazoan genes in the genome assembly of *P*. *laevis* as duplicated. This is close to the about 3% of duplicated metazoan BUSCO genes published before for monogonont rotifers like *B*. *calyciflorus* and *B*. *plicatilis* [146,147]. However, BUSCO classified 18.4% of the expected metazoan genes as duplicated, when run on the transcriptome-derived proteome of *P*. *laevis*. Different gene structures might have hampered the recognition of paralogues in the *P*. *laevis* genome. More likely, the presence of isoforms in the proteome led to an overestimation of the proportion of duplicates. Either way, also the increased value of 18.4% is much lower than corresponding estimates for bdelloids. In the *A*. *vaga* draft genome, for example, 73.4% of the metazoan genes were estimated to be duplicated, probably reflecting a tetraploid state [67,165]. Correspondingly, tetraploidy should have evolved on the bdelloid stem lineage or within bdelloids.

### Orthologous proteins in Monogononta, Bdelloidea, and Acanthocephala

**Fig 2:**
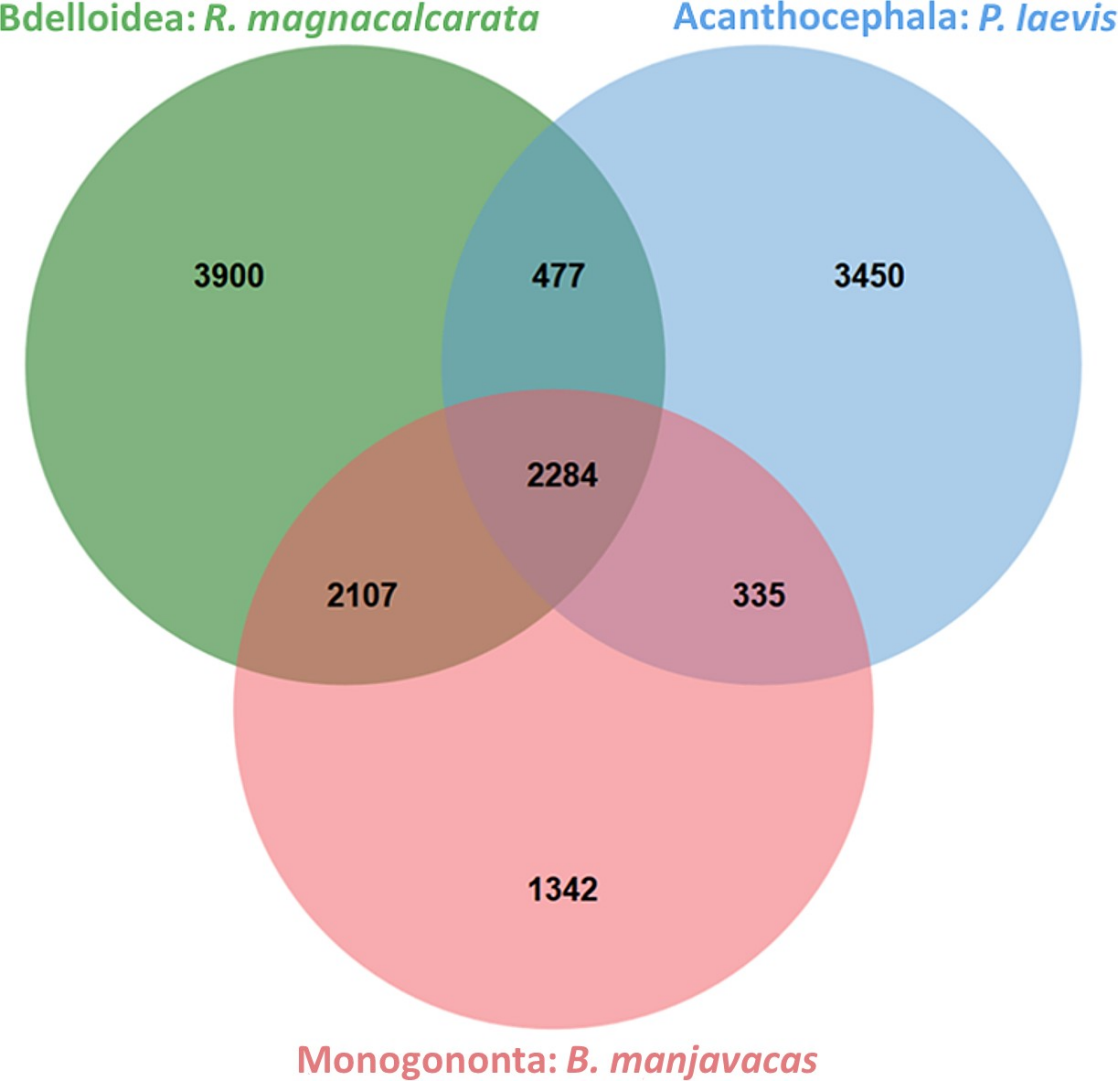
Venn diagram of orthologous protein clusters in the *B*. *manjavacas* (Monogononta), *R*. *magnacalcarata* (Bdelloidea), and *P*. *laevis* (Acanthocephala). The higher number of clusters shared between *R*. *magnacalcarata* and *P*. *laevis* than between the latter species and *B*. *manjavacas* is in accordance with the closer relationship of acanthocephalans to bdelloids than to monogononts (see main text). Also, the overall greater similarity of the monogonont and bdelloid proteomes to each other than to the more strongly derived *P*. *laevis* proteome is evident (compare Fig 3). The analysis was conducted with OrthoVenn1 using transcript-derived proteomes of the respective species. See S1 Text for an analogous analysis in an alternative species triple (no. 2). Transcriptomes of the monogonont and bdelloid were re-analyzed [130,131], while corresponding data for the acanthocephalan were newly generated in this study.

Based on approximately 35,000–36,000 proteins per species, OrthoVenn generated similar numbers of protein clusters for *B*. *manjavacas* (ca. 6,000) and *P*. *laevis* (ca. 6,500), while the corresponding number was increased in the bdelloid *R*. *magnacalcarata* (ca. 8,800) (Table 2). On the other hand, with slightly less than 60%, almost the same proportion of proteins was clustered in *R*. *magnacalcarata* and *P*. *laevis*, while the corresponding portion was of about half as large in *B*. *manjavacas*. In fact, there were 42% more clusters in the acanthocephalan-bdelloid than in the acanthocephalan-monogonont comparison. A likewise pattern was reproduced in an additional group of three species (S1 Text) and presumably reflects the increased gene content of bdelloids as tetraploids (see previous section) and their closer phylogenetic relationship to acanthocephalans than to monogononts [43,50–52]. Also, the higher number of clusters that *P*. *laevis* shares with *R*. *magnacalcarata* than with *B*. *manjavacas* (Fig 2) is consistent with the fact that Eurotatoria (Monogononta+Bdelloidea) is probably a paraphyletic taxon [43,50–56]. At the same time, the number of clusters shared by *P*. *laevis* with any of the (other) rotifers is lower than between the monogonont and the bdelloid. But such a pattern is actually to be expected considering that the coding sequences and the gene repertoire should be most derived in acanthocephalans.

**Fig 3:**
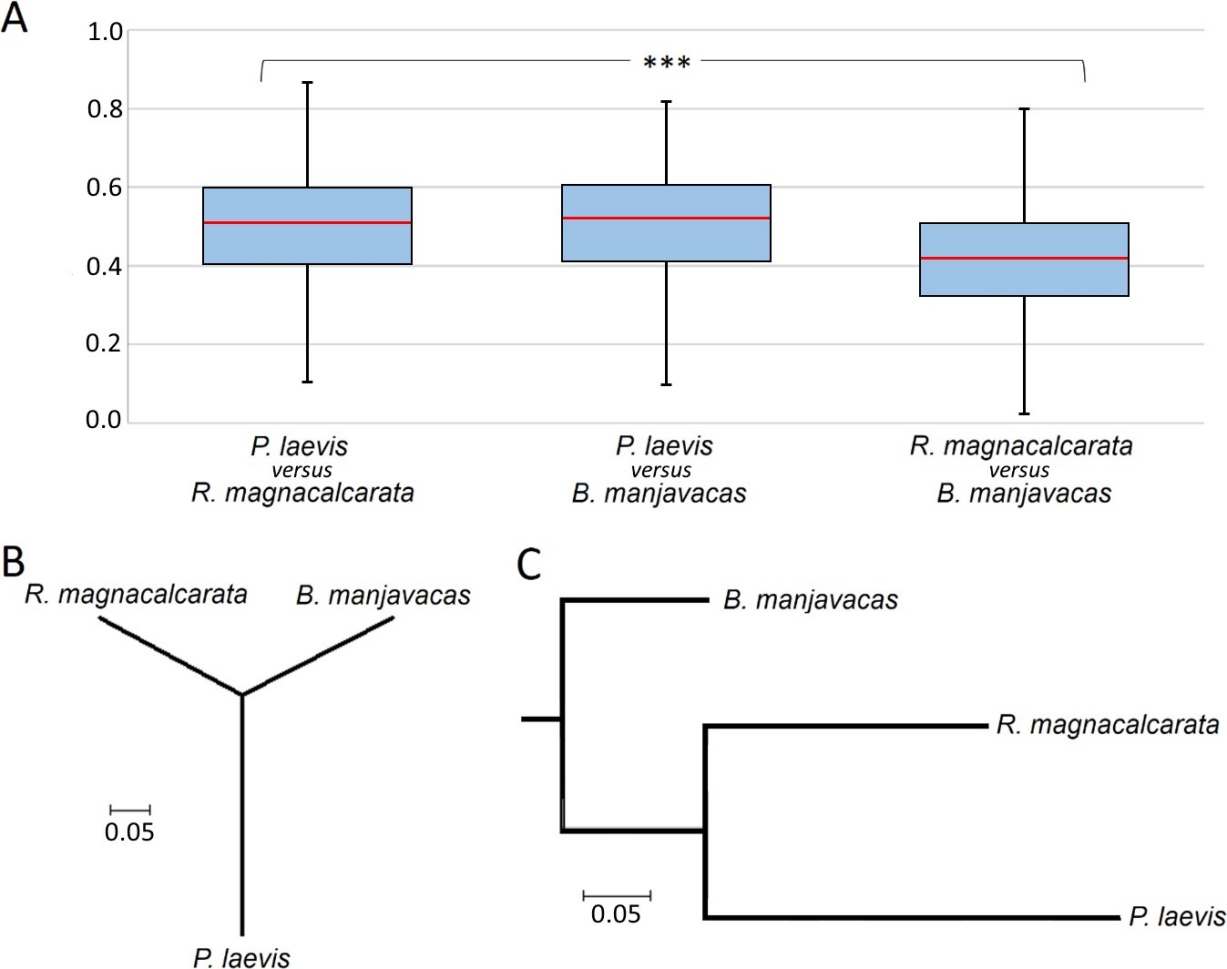
Pairwise comparisons of 750 orthologous single copy proteins between *B*. *manjavacas* (Monogononta), *R*. *magnacalcarata* (Bdelloidea), and *P*. *laevis* (Acanthocephala). **A)** Box plot diagram showing overall higher p-distances between orthologues from the acanthocephalan and any of the rotifers than between rotifer orthologues. The difference across all three pairs of comparison is significant at the level of p < 0.000 (***), as revealed by Kruskal-Wallis test (SPSS v. 23.0, IBM). Lower and upper boundaries of boxes with blue filling indicate 25th and 75th percentiles. Red horizontal bars correspond to median p-distances. Whiskers illustrate levels of the lowest and highest p-distance for each pair of comparison. Unrooted **(B)** and rooted **(C)** neighbor-joining tree demonstrating that acanthocephalan proteins evolved at increased rates. The phylogeny was drawn with MEGA X from median p-distances. Branch lengths give exchanges per amino acid. Transcriptomes of the monogonont and bdelloid were re-analyzed [130,131], while corresponding data for the acanthocephalan were newly generated in this study.

The protein clusters shared by all three species contained 752 clusters containing one orthologue from every species. After removal of highly variable sections, 750 of these single-copy clusters were used for evolutionary analyses. The corresponding p-distances were highest in the species pair *P*. *laevis*-*B*. *manjavacas* (median: 0.519), closely followed by the pair *P*. *laevis*-*R*. *magnacalcarata* (median: 0.511). Compared to this, the p-distances were clearly decreased, when inferred from orthologues of *B*. *manjavacas* and *R*. *magnacalcarata* (median: 0.420). Accordingly, Kruskal-Wallis test rejected the null hypothesis of equality with high significance (p < 0.000; Fig 3A). Neighbor-joining trees, built from median p-distances, illustrated that p-distances were primarily increased on the acanthocephalan lineage, while they were about the same for the other two lineages (Fig 3B and 3C). Thus, CDSs underwent significant reorganization in acanthocephalan evolution in particular, which accords with changes in acanthocephalan morphology, lifestyle, and physiology (e.g. [11,85,166]).

Enrichment analysis was revealing in another respect. Thus, seven out of thirteen GOs enriched in the protein clusters shared by the monogonont *B*. *plicatilis* and the acanthocephalan *P*. *laevis* indicated an involvement in dosage compensation of gene expression, while no such terms were found enriched in any of the protein clusters involving the bdelloid *R*. *magnacalcarata* (S8 Table). In fact, dosage compensation should be of particular importance in monogononts and acanthocephalans because these two form males and females [57,167]. While males are obligatory in acanthocephalans, they are not in monogononts. Instead, female monogononts react with the production of haploid males upon certain stimuli, followed by sexual reproduction and the formation of resting eggs [71]. In contrast, the relevance of dosage compensation, if it occurs, would be uncertain in bdelloids, for which no males have been reported and which are commonly believed to reproduce strictly parthenogenetic [57,67–69]. The generally increased complexity of the monogonont life history seems also to have left an imprint in the amount of GOs, which OrthoVenn found to be enriched in the clusters specific for *B*. *manjavacas* (S8 Table).

### HGT analysis

Approximately 35%, 36%, and 53% of the proteins in *P*. *laevis*, *B*. *manjavacas*, and *R*. *magnacalcarata*, respectively, were scrutinized for potential HGT. Following other investigators [68,131,168], we especially focused on potential HGT events from a non-metazoan. In *P*. *laevis*, the analytical pipeline described in the Materials and methods led to the 1,729 proteins (4.8%), which could be encoded by genes originating from non-metazoans. The application of the same pipeline to *R*. *magnacalcarata* resulted in 2,251 proteins (6.4%) that might be encoded by HGT candidates. Eyres et al. [131] calculated a very similar HGT rate of 5.7% for all transcripts of *R*. *magnacalcarata*, applying a slightly different approach. The same study reports even higher values of 7.0% and 7.6% for the genus *Rotaria*, which could reflect genome fragmentation and re-assembly in the course of repeated anhydrobiosis-hydrobiosis cycles [67]. In any case, the proportions of HGT candidates in bdelloids and *P*. *laevis* were clearly higher than in the monogonont *B*. *manjavacas*, where only 345 or 1% of all proteins were HGT candidates. Thus, the bdelloid and acanthocephalan were again more similar to each other than to the monogonont.

HGT from a metazoan cannot be ruled out in acanthocephalans, and it may even be regarded likely, considering that their close association with mandibulate and gnathostome hosts [1,3,50]. However, distinguishing whether the sequence similarity between metazoan genes is due to orthology or HGT is generally difficult. In any case, we found no evidence of an increased occurrence of HGT events from amphipod or teleost sequences in acanthocephalans. Rather, MEGABLAST comparison of protein sequences against the UniRef90 database (DIAMOND) led to similar amounts of best bit-score hits with either an amphipod or a teleost sequence, regardless of whether the query sequences originated from *P*. *laevis* or *R*. *magnacalcarata*. Consequently, the parasitic lifestyle seems not to promote the incorporation of host DNA into the genome of acanthocephalans, at least not in relation to bdelloids.

### The orange coloration of the *Pomphorhynchus* tegument

The tegument or epidermis makes up the major portion of the acanthocephalan body wall in acanthocephalans [169,170], and it is orange in *P*. *laevis* and its congeners. But metazoans usually do not possess genes for the biosynthesis of carotenoids, which likely account for the coloration of the *P*. *laevis* tegument. If such genes exist, they are supposedly acquired by gene transfer [171]. However, we did not find carotinoid biosynthesis genes amongst the HGT candidates in the *P*. *laevis* transcriptome. Nevertheless, we noticed GO terms relating to rhodopsin biosynthesis and ample connections to transmembrane receptors of the rhodopsin family in the Trinotate annotation (S7 Table). In addition, the GO terms of two of the OrthoVenn single-copy clusters referred to rhodopsin-specific enzymes, which were thus transcribed in the analyzed *P*. *laevis* specimens as well (S3 Table). Yet, rhodopsin contains the chromophore 11-cis-retinal, which is a carotinoid and to which there were also numerous connections in the Trinotate annotation (S7 Table). Thus, an enzymatic machinery for carotinoid processing apparently exists in acanthocephalans. A role of carotinoids in the acanthocephalan metabolism is additionally reflected by the fact that retinal is a derivative of vitamin A, which acanthocephalans are known to take up via surface [172]. Probably, the uptake of vitamin A and carotinoids happens along with lipids [15,173–175], which play a significant role in the nutrition of acanthocephalans, including *P*. *laevis* (see next section). However, if the orange coloration itself confers an adaptive benefit to the worms inside their gnathostome hosts remains elusive. A function as anti-oxidants would be conceivable. In contrast, orange coloration could protect cystacanths from UVB radiation and potentially increases their chances for host-transfer [176,177].

### The complex energy strategy of acanthocephalans

The above demonstrated reorganization of the entire acanthocephalan proteome also covered the energy metabolism. Thus, pairwise comparisons involving *P*. *laevis* led to higher p-distances and dN/dS values than pairwise comparisons between the monogonont and bdelloid. Notably, this was the case for 43 concatenated single-copy transcripts with special relevance for energy metabolism and also for their 532 without such commitment (Table 3). More revealing than the absolute values was the extent to which they differed, depending on the inclusion or exclusion of *P*. *laevis* in pairwise comparisons: In the case of energy-related genes, the respective factor was persistently higher for transcripts with energy-related GOs than for transcripts without such GOs (see parentheses in Table 3). Thus, energy-related transcripts and the genes behind did not only accumulate, in relative terms, particularly many nucleotide substitutions on the lineage to *P*. *laevis*. Rather, these changes included disproportionally many non-synonymous exchanges (dN). This suggests an increased proportion of adaptively evolving amino acid positions in the energy-related proteome of acanthocephalans.

**Table 3:** Pairwise comparisons of concatenated transcripts sorted after their function.

Protein class	*P*. *laevis* vs. *R*. *magnacalcarata*	*P*. *laevis* vs. *B*. *manjavacas*	*B*. *manjavacas* vs. *R*. *magnacalcarata*
Energy-related [p-distance]	0.465 (1.275)	0.468 (1.283)	0.365 (not applicable)
Not energy-related [p-distance]	0.493 (1.218)	0.497 (1.229)	0.405 (not applicable)
Energy-related [dN/dS]	0.004 (1.333)	0.005 (1.667)	0.003 (not applicable)
Not energy-related [dN/dS]	0.005 (1.250)	0.006 (1.500)	0.004 (not applicable)

Values in parenthesis give fold-changes in relation to the species pair in the right column. dN/dS, rate ratio of non-synonymous to synonymous substitution rates.

**Fig 4:**
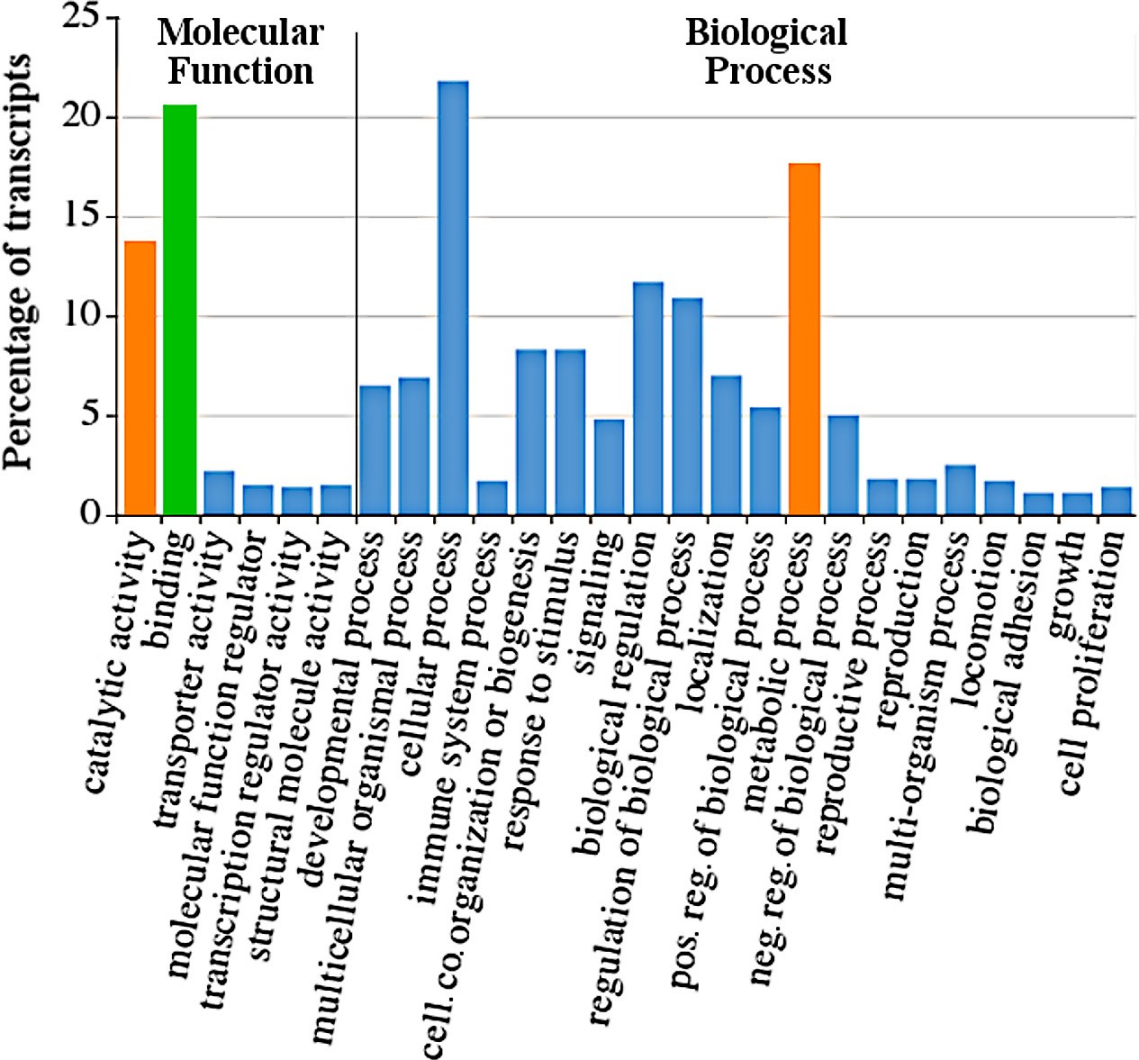
Functional annotation of the *P*. *laevis* proteome. Shown are second level Gene Ontology (GO) terms represented by at least 1% of the transcripts. GO terms relating to catalytic activity and binding (Molecular Function) and metabolic processes (Biological Process) belong to the most abundant ones. Orange highlights cata- and metabolic functions; green denotes involvement in binding. GO term analysis was carried out with the aid of the Trinotate pipeline. Visualization was done with WEGO and GIMP 2.8.20. The less revealing subdivision of GOs under the parent term cellular component is not shown. *cell*. *co*., cellular component; *neg*. *reg*., negative regulation; *pos*. *reg*., positive regulation.

A closer look for the GOs underlined the importance of energy metabolism for *P*. *laevis*. Thus, GOs relating to catabolism and metabolism were clearly amongst the most abundant ones (see orange bars in Fig 4). The same chart shows numerous transcripts associated with the GO term “binding”. In the case of an acanthocephalans like *P*. *laevis*, these could include a lot of transcripts involved in nutrient uptake via the surface.

**Fig 5:**
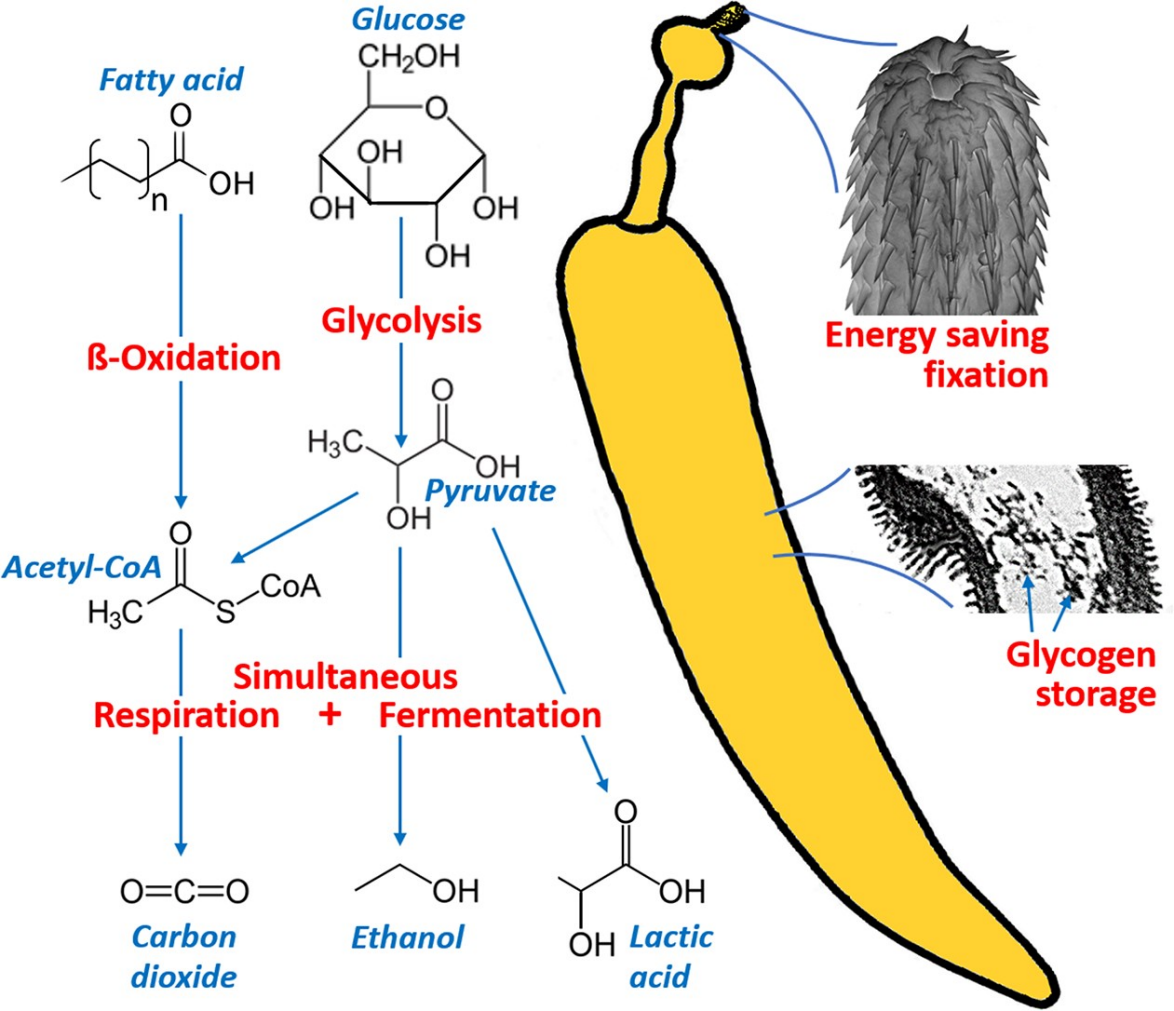


Inspection of the individual GOs revealed additional insight in the complexity of energy metabolism in *P*. *laevis*. Amongst others, Trinotate associated many *P*. *laevis* transcripts directly (without parent terms) with corresponding GO terms (S7 Table), including genes like Fat storage-inducing transmembrane protein and Long-chain fatty acid transport protein 4. In addition, there were many GOs referring to coenzyme A, which is commonly known to transfer carbons into the Krebs cycle. *Pomphorhynchus laevis* should additionally be capable of lactic acid fermentation (Fig 5), just as reported for *Moniliformis dubius* [178]. However, with only a single *P*. *laevis* transcript pointing to such an engagement (see S7 Table: D-lactate dehydrogenase), the pathway should be of less significance than in platyhelminths, which have a high number of lactate dehydrogenase genes [73]. Furthermore, the annotation of acetyl-coenzyme A synthetase could point to the excretion of acetate, which before was observed in the archiacanthocephalan *M*. *dubius* [178] and other parasitic taxa [179].

Functional diversification through alternative splicing and neo- and subfunctionalization of gene copies is generally considered selectively advantageous [180,181]. Accordingly, the investigation of protein clusters with regard to functional characteristics should be particularly elucidating. In line with this postulate, all nine GO terms enriched in the clusters that OrthoVenn had built from the *P*. *laevis* proteome were related to energy metabolism (S8 Table). In contrast, only a single GOs appeared to be enriched in analogous analyses of species from Monogononta and Bdelloidea (S8 Table: acyl-CoA dehydrogenase activity, S9 Table: none). In the *P*. *laevis* proteome, some of the protein clusters refer to aerobic energy metabolism, subsumed under cellular respiration and electron transport chain (Fig 5). With pyruvate metabolic process and pyruvate dehydrogenase (NADP+) activity two additional GO terms stressed the connection of oxygen-consuming and oxygen-independent energy metabolism. In fact, pyruvate is well known as the product of anaerobic glycolysis, from which two carbons are transferred into the Krebs cycle [182]. However, the educt of pyruvate production, phosphoenolpyruvate, in acanthocephalans also connects to fermentation [166,183,184]. The importance of fermentation for the acanthocephalan energy metabolism was additionally reflected by the *P*. *laevis*-specific enrichment of the GO terms acetaldehyde dehydrogenase (acetylating) activity and alcohol dehydrogenase (NAD) activity (S8 Table). This agrees with earlier findings that acanthocephalans ferment glycogen rapidly to ethanol [166,184,185]. Together, the different functional implications suggest that one of the acanthocephalan responses to the challenge of reduced intestinal oxygen tension is to use the scarce oxygen for efficient ATP production and, at the same time, to engage in less-effective fermentation pathways (Fig 5). This strategy seems to be obligate to acanthocephalans, which reportedly maintain fermentation in vitro even when oxygen is present [178,186,187].

A second answer to the challenge of the high energy demand in an oxygen-depleted environment manifests in the acanthocephalan capability to store considerable amounts of glycogen [188,178] (Fig 5). Averaged over the entire body glycogen quantities of up to 3.7% wet weight were measured in single individuals and mean values in cohorts of worms reached up to 2.3% [186,189]. Especially, in the tegument of acanthocephalans and especially in the musculature, glycogen is deposited in such high quantities that aggregated glycogen particles are already visible at low magnification (Fig 1 in [188]; Fig 5A in [11]). Glycogen storage also appears to have a correlate in the acanthocephalan transcriptome of *P*. *laevis*. Thus, some of the annotated proteins are involved in glycogen synthesis and glycogen decomposition, thereunder glycogen [starch] synthase and glycogen phosphorylase 1 (S7 Table). Notably, glycogen phosphorylase was lately considered a potential target for the control of other parasitic helminths [73]. According to the present results, the pathway could also hold targets for the control of acanthocephalans in cultures of fish [14,35,190].

A third aspect ensuring sufficient energy supply to reproduction is that thorny-headed worms, like other intestinal parasites [7], attach themselves to the intestinal wall of the vertebrate host. The development of the respective attachment organ, the proboscis, should be costly, but the benefits obviously outweigh the costs [191]. In fact, the attachment causes lesions to the intestinal wall, so that acanthocephalans can absorb nutrients and also oxygen from the incoming blood and decaying tissue [15,192]. However, the attachment also frees the worms from the need to counteract the risk of dislodgement through energy-consuming movements (see [11,12,191]). In this context, it is striking that parasitic helminths were generally said to need oxygen only for their movement, but not for survival [193]. In fact, *P*. *laevis* can even serve as an extreme example of fixed anchoring within parasitic helminths: In the course of anchoring, the anterior body pole reaches the outside of the intestinal wall, followed by the formation of a subterminal dilatation, which then prevents the anterior end from sliding back into the intestine [20]–or, as Müller (1776) put it with reference to the original investigator J. Zoega: “*Ech*. *laevis* proboscide echinata, pone apicem in sphaeram laevem dilatata.” There is actually not much to add, except perhaps that the eponymous left-sidedness does not seem to be a general pattern.

## Conclusions

We have assembled the first draft of an acanthocephalan nuclear genome, in addition to the first transcriptome assembly. Since sex determination in *Pomphorhynchus* likely follows an X0 system [194], the draft genome should be representative for both sexes of *P*. *laevis*. With a total span of ca. 260 Mb, its size is within the range reported for bdelloids [67,68] but larger than draft genomes in monogononts [144–147,149]. In addition, the repetitive portion of the *P*. *laevis* genome (63%) is higher than corresponding values published for monogononts and bdelloids [68,146]. Furthermore, there was no evidence for a distinct reduction of the non-repetitive portion in *P*. *laevis*, which one might expect for an obligate parasite. The number of transcripts and the encoded proteins was in the range of the corresponding counts in free-living monogonont and bdelloid rotifers [130,131]. Nevertheless, in a larger phylogenetic context, the gene repertoire seems to have experienced a reduction in acanthocephalan evolution. Thus, we detected only 73% of the expected metazoan genes in the *P*. *laevis* transcriptome, a dimension that was reported also for parasitic helminths [161,162]. The reduction in gene repertoire was also reflected in a reduced complement of proteins regulating development, as expected for a parasite with comparably simple body organization [159]. In fact, the reduced digestive tract of acanthocephalans [42] coincided with the non-detection of *hox1* and *hox2* in *P*. *laevis*, which in other Spiralia control the differentiation of the stomatogastric nervous system [155,164]. Yet, if *P*. *laevis* and acanthocephalans in general really lack anterior *hox* genes, the ganglion inside the receptacle might correspond to the mastax ganglia in other gnathiferans [195].

The genes and proteins retained obviously experienced significant reorganization in acanthocephalan evolution. Thus, we found protein distances to be higher between *P*. *laevis* and either *B*. *manjavacas* or *R*. *magnacalcarata* than between the latter two. At the same time, the bdelloid and monogonont shared more orthologous gene clusters than each of them with the acanthocephalan *P*. *laevis*–a pattern that we reproduced in an alternative species triple (no. 2). Probably, the higher similarity of monogonont and bdelloid proteomes reflects that the LCAs of both taxa as well as the species analyzed maintained the plesiomorphic condition of free-living. In contrast, the more dissimilar proteome of *P*. *laevis* likely reflects significant changes in acanthocephalan evolution towards an endoparasitic two-host cycle [3,7,45,50,51].

The present findings additionally suggest that HGT from non-metazoans is common in the Rotifera-Acanthocephala clade. Thus, about 4.8–6.4% of the transcripts of *R*. *magnacalcarata* and *P*. *laevis* were found to be of potential non-metazoan origin, while the respective rate in *B*. *manjavacas* was only about 1%. In the stem line of Acanthocephala, elevated HGT may have laid the grounds for the establishment of an endoparasitic lifestyle as has been similarly postulated for animal parasitizing nematodes [72,73], phytopathogenic nematodes [63,74,75], and parasitic plants from Orobanchaceae [76]. In fact, HGT might endow parasites with new capabilities that open up new paths in their evolution [72,73].

In the GO annotation of genes or proteins, there were numerous indications of processing of carotenoids, which are probably taken up via surface together with lipids [172]. In addition, analyses of GOs together with previously published data from physiological measurements suggest a complex energy strategy of acanthocephalans. Firstly, the scarce oxygen available in the vertebrate digestive tract is obviously used for ATP production via respiration, but at the same time less effective fermentation occurs. In line with this, we found transcripts related to energy metabolism to be more derived in *P*. *laevis* than in the monogonont and bdelloid species studied. We additionally provide first-time evidence for the existence of aerobic and anaerobic metabolic processes in acanthocephalans (compare [166,178,183–185]). Secondly, in accordance with histological data (Fig 1 in [188]; Fig 5A in [12]), the acanthocephalan ability to store large amounts of glycogen was reflected in the annotations. Thirdly, it is clear from the mode of their anchoring that acanthocephalans reduce the energy required to remain inside the digestive tract [11,12,191]. While an attachment is common in endoparasites and also occurs in tapeworms (Platyhelminthes, Cestoda) and flukes (Platyhelminthes, Trematoda) [7], the precise mode of attachment displayed by *Pomphorhynchus* specimens is quite special: After the hooked proboscis has pierced the intestinal wall of the vertebrate host, the anterior neck widens to a plug-like dilatation holding the worm in position [20]. This extreme form of anchoring may have been favorable to uncover peculiarities in the energy metabolism. In any case, it should be revealing if the patterns described will persist when including species of Seisonidea, a closely related taxon living on marine crustaceans (Crustacea, Leptostraca), into the comparison [45,49–51,196,197].

## Supporting information

S1 Table Megablast_nuclear genome vs. custom carp database. The *P*. *laevis* genome assembly was blasted against a custom database (PRJNA352247, AP009047.1, GFWU01000001-GFWU01049434) with NCBI BLAST+ (E-value ≤ 1e-05; identity ≥ 85%).
(XLSX)

S2 Table Megablast_transcriptome vs. custom carp database. The *P*. *laevis* transcriptome assembly was blasted against a custom database (PRJNA352247, AP009047.1, GFWU01000001-GFWU01049434) with NCBI BLAST+ (E-value ≤ 1e-05; identity ≥ 85%).
(XLSX)

S3 Table OrthoVenn_single-copy cluster classification. Classification into clusters with direct involvement in energy metabolism, clusters without such an involvement, and clusters with uncertain classification or lacking an annotation.
(XLSX)

S4 Table GMAP_transcripts mapped to the masked nuclear genome assembly. (XLSX)

S5 Table Blastp-blastx_transcripts not mapped to the masked nuclear genome assembly. (XLSX)

S6 Table tRNAscan-SE_annotation report. (XLSX)

S7 Table Trinotate_annotation report. (XLSX)

S8 Table OrthoVenn_gene ontologies enriched in species triple 1. Analysis including the monogonont *B*. *manjavacas* and the bdelloid *R*. *magnacalcarata*, besides *P*. *laevis*.
(XLSX)

S9 Table OrthoVenn_gene ontologies enriched in species triple 2. Analysis including the monogonont *B*. *calyciflorus* and the bdelloid *A*. *ricciae*, besides *P*. *laevis*.
(XLSX)

S1 Text More details on materials, methods, results, and discussion. (DOCX)
