# Supplementary material for: The genome, transcriptome, and proteome of the fish parasite Pomphorhynchus laevis (Acanthocephala)
Source: PLoS One. 2020 Jun 23;15(6):e0232973. doi: 10.1371/journal.pone.0232973 (PMC7310846; doi:10.1371/journal.pone.0232973)
Supplement: S1 Text — (DOCX) [file pone.0232973.s010.docx]

# **S.1 Text. More details on materials, methods, results, and discussion**

**Contents**

**1** [**GenomeScope analysis 2**](#_Toc11674852)

**2** [**Genome assembly 3**](#_Toc11674853)

**3** [**De novo repeat assembly with dnaPipeTE 3**](#_Toc11674854)

**4** [**Classification of *P. laevis* repeats by RepeatMasker 5**](#_Toc11674855)

**5** [**OrthoVenn analysis for second species triple 6**](#_Toc11674856)

**6** [**Horizontal gene transfer (HGT) 10**](#_Toc11674859)

**7** [**References 11**](#_Toc11674860)

##

## **1 GenomeScope analysis**

Genome size of *Pomphorhychus laevis* was estimated from Illumina reads on the basis of k‑mer counting (k = 21) using Jellyfish [1]. The GenomeScope [2] online tool calculated a genome size of 265 to 281 Mb. With a total number of about 6,000,000 (= 6e+06), the k‑mers occurring ca. 44 times in the dataset made up the largest fraction, as illustrated by the according global peak in the GenomeScope graph (Fig A in this document). A second peak at lower coverage represents polymorphic k-mers. The polymorphic proportion of the *P. laevis* specimen analyzed was estimated at 0.22% of the haploid genome. GenomeScope gave an estimate of 45 to 51% (127 to 137 Mb) to be repetitive. This percentage is smaller than calculated for the genome assembly with RepeatMasker (ca. 60%), but still exceeds the corresponding values reported for nuclear genome assemblies of monogonont and bdelloid rotifers (see Results and discussion in the main document). Lastly, the proportion of faulty reads was 0.067% according to GenomeScope.


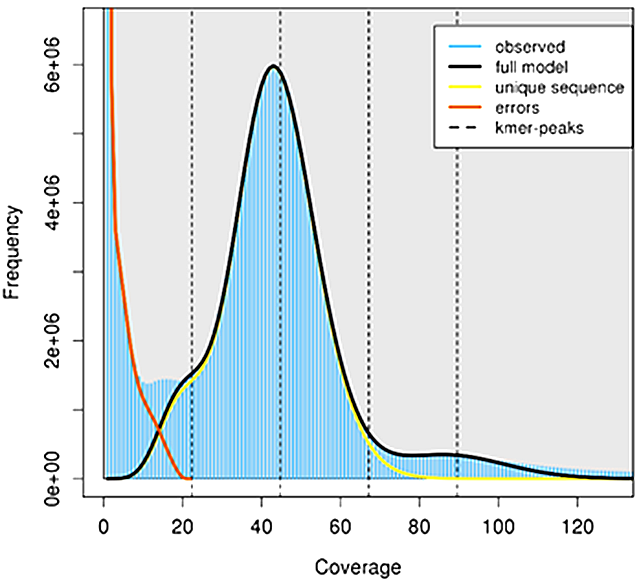


**Fig A. GenomeScope analysis of the Illumina *P. laevis* data (k = 21).**

## **2 Genome assembly**

To create the *P. laevis* draft genome, we used several assembly programs. The metrics of the individual assemblies are listed in Table A. The description of the assembly steps is contained in the Material and methods section of the main document.

**Table A. Assembly metrics from different assembly steps and the final genome draft.**

| **Metric** | **Canu** | **Platanus** | **DBG2OLC** | **Final assembly** |
| --- | --- | --- | --- | --- |
| Span (bp) | 244,225,086 | 332,418,352 | 255,291,314 | 260,273,928 |
| No. contigs | 5,739 | 1,678,495 | 4,601 | 4,021 |
| Contig N50 [bp] | 58,612 | 331 | 100,044 | 126,104 |
| Mean contig length [bp] | 42,555.34 | 198.05 | 55,486.05 | 64,739.17 |
| Longest contig [bp] | 320,527 | 30,982 | 843,289 | 844,615 |
| Shortest contig [bp] | 1,159 | 69 | 59 | 1,137 |

## **3 De novo repeat assembly with dnaPipeTE**

The program dnaPipeTE [3] uses only a subset of reads for a Trinity assembly [4], which should give an estimate of the composition of the repetitive areas. The subset is determined by setting an estimated genome size and a coverage. We used a pipeline based on Schell and others [5] for dnaPipeTE. For all runs, we assumed a genome size of 250 Mb for *P. laevis*. In addition, only trimmed and filtered forward Illumina reads were used (see Materials and methods in main document). The minimum cutoff for contigs was set to 50 bp. We implemented coverages from 0.001 to 0.500, in order to determine the best fitting one for repeat assembly. In doing so, we found that N50 values were lowered towards the tails of the distribution (Fig B in this document). The second-highest N50 value (275), which was only slightly smaller than the global optimum, was generated for an acceptable coverage value (0.010). As also the number of contigs assembled (878) appeared acceptable we selected the coverage of 0.010 for further analyses. Because dnaPipeTE is a heuristic program, we carried out 50 runs with the best-fitting coverage.


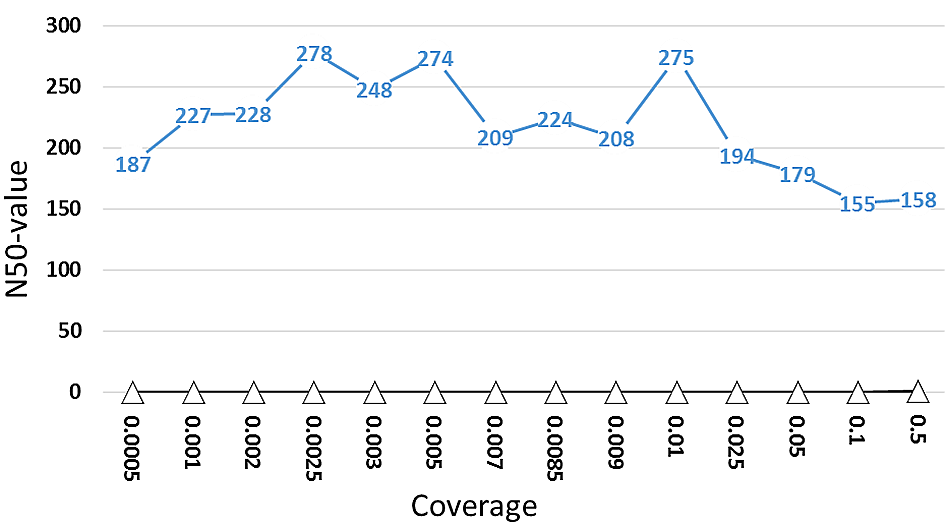


**Fig B. N50 values of 15 dnaPipeTE runs with different coverage depths.**

The dnaPipeTE contigs of all 50 runs, which matched in 90% of their positions, were grouped into 15,675 clusters with CD-HIT-EST [6]. The processed Illumina reads for one lane (L002) were mapped with the BWA mem algorithm of BWA v.0.7.15 [7] to the main sequences of each cluster. We filtered out sequences smaller than 100 bp and sequences that showed an average coverage < 123. We assumed a minimal coverage of 123 because it equals three times the average of the genome coverage from Illumina reads L002 (see Material and Methods in main document). This should account for potential unequal representation of genome sections in the read subsets. We annotated the remaining 9,255 sequences with TEclass [8].

## **4 Classification of *P. laevis* repeats by RepeatMasker**

**Table B. Classification of repeats contained in the *P. laevis* draft genome**.

| **Level 1** | **Level 2** | **Count** | **Base pairs masked** | **Percentage masked** |
| --- | --- | --- | --- | --- |
| DNA | total | 61,252 | 7,707,447 | 2,94% |
|  | Merlin | 1,641 | 733,553 | 0,28% |
|  | hAT-Charlie | 1,419 | 641,856 | 0,25% |
|  | other | 58,192 | 6,332,038 | 2,41% |
| LINE | total | 220,132 | 93,740,011 | 36,01% |
|  | Penelope | 55,473 | 16,593,383 | 6,38% |
|  | CRE-II | 23,496 | 12,410,242 | 4,77% |
|  | L2 | 13,879 | 12,153,808 | 4,67% |
|  | CR1 | 17,878 | 10,801,921 | 4,15% |
|  | Jockey | 9,995 | 10,676,796 | 4,10% |
|  | RTE-BovB | 11,912 | 5,941,008 | 2,28% |
|  | RTE-X | 2,835 | 1,532,276 | 0,59% |
|  | Tad1 | 826 | 707,340 | 0,27% |
|  | Rex-Babar | 1,061 | 609,835 | 0,23% |
|  | R1 | 544 | 562,034 | 0,22% |
|  | other | 82,233 | 21,751,368 | 8,35% |
| LTR | total | 49,877 | 14,411,931 | 5,54% |
|  | Gypsy | 7,090 | 4,895,549 | 1,88% |
|  | DIRS | 324 | 251,826 | 0,10% |
|  | other | 42,463 | 9,264,556 | 3,56% |
| Unknown |  | 196,525 | 42,168,859 | 16,20% |
| SINE |  | 11,526 | 1,001,247 | 0,38% |
| Total interspersed |  | 539,318 | 159,030,169 | 61,10% |
| Low_complexity |  | 13,171 | 767,092 | 0,29% |
| Simple_repeat |  | 63,567 | 4,070,655 | 1,56% |
| rRNA |  | 157 | 387,672 | 0,15% |
| Total |  | 616,213 | 164,255,588 | 63,11% |

## **5 OrthoVenn analysis for second species triple**

A second OrthoVenn analysis was carried out with *P. laevis* and two alternative representatives of bdelloid and monogonont rotifers. We selected *Adineta ricciae* as a representative for Bdelloidea, for which transcriptome data were published before [9]. With this choice, the second species trio contained not only a different species, but also a different genus of Bdelloidea than the first one, which included *Rotaria magnacalcarata* (see main document). For Monogononta, only transcriptome data of *Brachionus* species were available at the time of the study. Thus, we chose a corresponding dataset of *Brachionus calyciflorus* [10] for second OrthoVenn analysis. The transcripts of *A. ricciae* and *B. calyciflorus* were transformed into proteins using TransDecoder with the same settings as used for *P. laevis*. Only the single best protein was issued for each transcript and all proteins had to span at least 30 amino acids (see Material and methods in main document). We chose the metazoan database, uploaded the protein sets of *P. laevis*, *A. ricciae and B. calyciflorus*, and ran OrthoVenn with default settings.

The bdelloid *Adineta ricciae* showed less transcripts and proteins than *R.* *magnacalcarata* (compare Table 2 in main document and Table C below)*.* However, this is not necessarily a valid pattern because only those transcripts were published for *A. ricciae*, for which E-values of BLAST hits in the UniProtKB database were ≤ 10-5 [9]. Thus, the transcriptome used for *A. ricciae* was probably not as complete as the one representing *R.* *magnacalcarata*. Likewise, the *B. calyciflorus* dataset had less transcripts and proteins encoded than the corresponding dataset for *B. manjavacas*, which was the monogonont representative in the first species triple. This again could reflect differences in the pipeline used for transcriptome analysis but differences in life histories might also have contributed [10,11].

**Table C.** T**ranscriptome and proteome metrics of P. laevis and alternative bdelloid and monogonont rotifers.**

| **Key parameters**  **of assemblies** | **Monogononta:**  ***B. calyciflorus**** | **Bdelloidea:**  ***A. ricciae**** | **Acanthocephala:**  ***P. laevis***** |
| --- | --- | --- | --- |
| Transcriptome span (bp) | 21,417,057 | 15,704,640 | 33,776,651 |
| No. contigs | 33,088 | 28,922 | 42,888 |
| Contig N50 (bp) | 825 | 646 | 1,374 |
| Mean contig length (bp) | 647 | 543 | 788 |
| Longest contig (bp) | 7,851 | 3,674 | 15,909 |
| Shortest contig (bp) | 200 | 161 | 200 |
| No. proteins | 29,973 | 28,729 | 35,622 |
| Proteome span (AS) | 5,480,367 | 4,632,777 | 7,059,720 |
| Mean protein length (AS) | 183 | 161 | 198 |
| No. OrthoVenn cluster  (included proteins %) | 7,225  (42.1%) | 8,559  (62.6%) | 6,481  (58%) |

*According to [9,10]; **Newly generated data.

In this second OrthoVenn analysis, as in the first, 58% of the *P. laevis* proteins were assigned to clusters (compare Table 2 in main document and Table C above). In further agreement with first OrthoVenn results, a similar proportion of proteins could be grouped into clusters in case of the bdelloid representative (62.6%), while the corresponding proportion was decreased in the monogonont (42.1% see Table C above). As already-mentioned in the Results and discussion section (main document), this pattern could reflect a closer phylogenetic relationship of bdelloids to acanthocephalans than to monogononts [12–15]. Notwithstanding the wide correspondence of OrthoVenn analyses in both species triples, single counts differed (compare Figure 2 in main document and Fig C below). In addition, less GO terms were found enriched in the protein clusters specific to the bdelloid representative than in the clusters specific to the monogonont representative (see S.8 Table). As mentioned in the main document, this could reflect a more complex life history in monogononts.


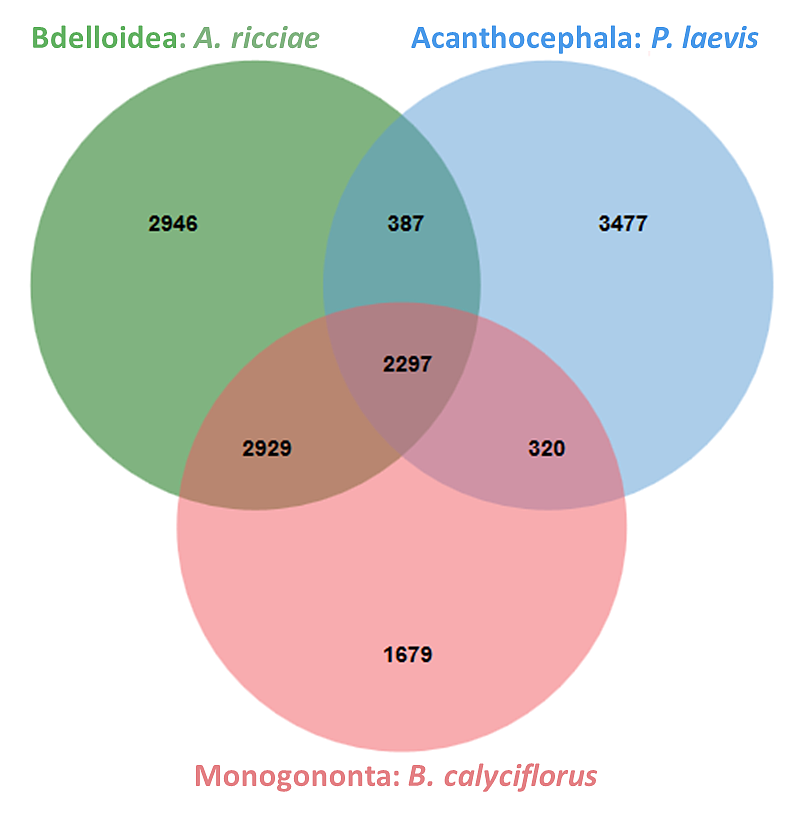


**Fig C.** **Venn diagram of orthologous protein clusters from *B. calyciflorus*, *A. ricciae*, and *P. laevis*; generated with OrthoVenn.**

According to OrthoVenn, both species triples consistently share about 35% of the protein clusters (compare Fig 2 in main document and Fig C above). Analysis of the second species trio additionally confirmed that *P. laevis* shares more orthologous protein clusters with the bdelloid species than with the monogonont species. As already discussed in the main document, this could reflect the closer phylogenetic relation of bdelloids to acanthocephalans than monogononts. In extension of this argument, we ascribe the even higher share between the monogonont and the bdelloid as representing the plesiomorphic condition. As outlined in the Results and discussion (main document), the proteome of *P. laevis* underwent a significant reorganization regarding its composition and the sequences of individual proteins. Both factors imply that there should be less consistency between the proteomes of the acanthocephalan on the one hand and monogononts and bdelloids on the other hand than between species of the latter two taxa.

Furthermore, second OrthoVenn analysis reproduced an enrichment of GOs referring to energy metabolism, such as “pyruvate metabolic process” and “pyruvate dehydrogenase (NADP+) activity”, in the protein clusters specific to *P. laevis* (compare Results and discussion in main document). However, the number of GO terms enriched in *P. laevis*-specific clusters was increased. Many of these GOs can be attributed to cell differentiation (e.g. “lymph gland crystal cell differentiation”, “embryonic crystal cell differentiation”, “R8 cell fate commitment”) and anatomic structure development (e.g. “lymph gland development“, “negative regulation of compound eye photoreceptor development“, “regulation of R8 cell spacing in compound eye”). The *P. laevis* data used for both OrthoVenn analyses were sequenced from a pool of adult males and females (containing eggs) and juvenile individuals. It is thus possible that these GO terms only appear in the second analysis to be enriched because the datasets of *B. calyciflorus* and *A. ricciae* were less representative for the entire lifecycle of the respective species, than it is the case in the analyzed transcriptome data of *B. manjavacas* and *R. magnacalcarata*. In support of such possibility, the *B. manjavacas* transcriptome data represented different stages of development due to a repeated sampling from a single culture at different times [11], while the analyzed *B. calyciflorus* strain was sampled only once. In addition, the *A. ricciae* transcriptome analyzed is presumably less comprehensive due to a stricter filtering. Accordingly, the *P. laevis* transcriptome, compared to the data representing *B. calyciflorus* and *A. ricciae*, could be disproportionally comprehensive. The limitations in the comparability of the datasets of *B. calyciflorus*, *A. ricciae*, and *P. laevis* could also explain that GOs referring to dosage compensation did not reappear in second OrthoVenn analysis. After all, the better comparability let us focus on the species triple *B. manjavacas*, *R. magnacalcarata*, and *P. laevis* in the main document.

## **6 Horizontal gene transfer (HGT)**

The proteins of *B. manjavacas* [11], *R. magnacalcarata* [16], and *P. laevis*, as derived from transcripts, were analyzed for potential non-metazoan horizontal gene transfer (HGT) (see main document). We present the metrics in Table D below.

**Table D. HGT analysis metrics.**

| **Parameter** | ***B. manjavacas**** | ***R. magnacalcarata**** | ***P. laevis***** |
| --- | --- | --- | --- |
| TransDecoder proteins | 35,747 | 35,161 | 35,622 |
| Proteins with DIAMOND hits to Uniref90 (E-value ≤ 1e-05) | 22,202 | 19,288 | 12,354 |
| HGT results^§^ | 12,728 | 18,796 | 12,343 |
| HGT candidates (HGT index > 30;  consensus hit support > 90%) | 395 | 2,280 | 1,934 |
| HGT candidates,  filtered for potential contamination | 345 | 2,251 | 1,726 |
| HGT share | 1% | 6,4% | 4.8% |

*According to [11,16]; **Newly generated data; ^§^Excluding hits referring to rotifers or acanthocephalans (see text above).

By setting “taxid_skip” to rotifers or acanthocephalans, we skipped hits to closely related organisms for the inference of HGT index and consensus hit support. Therefore, not all proteins with DIAMOND hits were regarded as evidence for HGT. For example, most proteins from *B. manjavacas*, 62.1%, showed a hit with an E-value ≤ 1e-05 to the Uniref90 database. However, many of these hits actually referred to *Brachionus plicatilis* and thus were not considered as HGT candidates.

**7 References**

1. Marçais G, Kingsford C. A fast, lock-free approach for efficient parallel counting of occurrences of k-mers. Bioinformatics. 2011;27: 764–770.

2. Vurture GW, Sedlazeck FJ, Nattestad M, Underwood CJ, Fang H, Gurtowski J, et al. GenomeScope: fast reference-free genome profiling from short reads. Bioinformatics. 2017;33: 1–3.

3. Goubert C, Modolo L, Vieira C, Moro CV, Mavingui P, Boulesteix M. *De novo* assembly and annotation of the Asian tiger mosquito (*Aedes albopictus*) repeatome with dnaPipeTE from raw genomic reads and comparative analysis with the yellow fever mosquito (*Aedes aegypti*). Genome Biol Evol. 2015;7: 1192–1205.

4. Grabherr MG., Haas BJ, Yassour M, Levin JZ, Thompson DA, Amit I, et al. Full-length transcriptome assembly from RNA-Seq data without a reference genome. Nat Biotechnol. 2011;29: 644–652.

5. Schell T, Feldmeyer B, Schmidt H, Greshake B, Tills O, Truebano M, et al. An annotated draft genome for *Radix auricularia* (Gastropoda, Mollusca). Genome Biol Evol. 2017;9: 585–592.

6. Huang Y, Niu B, Gao Y, Fu L, Li W. CD-HIT Suite: a web server for clustering and comparing biological sequences. Bioinformatics. 2010;26: 680–682.

7. Li H. Aligning sequence reads, clone sequences and assembly contigs with BWA-MEM. Available at arXiv:1303.3997.

8. Abrusan G, Grundmann N, DeMester L, Makalowski W. TEclass--a tool for automated classification of unknown eukaryotic transposable elements. Bioinformatics. 2009;25: 1329–1330.

9. Boschetti C, Carr A, Crisp A, Eyres I, Wang-Koh Y, Lubzens E, et al. Biochemical diversification through foreign gene expression in bdelloid rotifers. PLOS Genet. 2012;8: e1003035.

10. Hanson SJ, Stelzer C-P, Welch DBM, Logsdon JM. Comparative transcriptome analysis of obligately asexual and cyclically sexual rotifers reveals genes with putative functions in sexual reproduction, dormancy, and asexual egg production. BMC Genomics. 2013;14: 412.

11. Gribble KE, Mark Welch DB. Genome-wide transcriptomics of aging in the rotifer *Brachionus manjavacas*, an emerging model system. BMC Genomics. 2017;18: 1–14.

12. Wey-Fabrizius AR, Herlyn H, Rieger B, Rosenkranz D, Witek A, Welch DBM, et al. Transcriptome data reveal syndermatan relationships and suggest the evolution of endoparasitism in Acanthocephala via an epizoic stage. PLOS One. 2014;9: e88618

13. Sielaff M, Schmidt H, Struck TH, Rosenkranz D, Mark Welch DB, Hankeln T, et al. Phylogeny of Syndermata (syn. Rotifera): Mitochondrial gene order verifies epizoic Seisonidea as sister to endoparasitic Acanthocephala within monophyletic Hemirotifera. Mol Phylogenet Evol. 2016;96: 79–92.

14. Witek A, Herlyn H, Meyer A, Boell L, Bucher G, Hankeln T. EST based phylogenomics of Syndermata questions monophyly of Eurotatoria. BMC Evol Biol. 2008;8: 345.

15. Garey JR, Near TJ, Nonnemacher MR, Nadler SA. Molecular evidence for Acanthocephala as a subtaxon of Rotifera. J Mol Evol. 1996;43: 287–292.

16. Eyres I, Boschetti C, Crisp A, Smith TP, Fontaneto D, Tunnacliffe A, et al. Horizontal gene transfer in bdelloid rotifers is ancient, ongoing and more frequent in species from desiccating habitats. BMC Biol. 2015;13: 90.
